# Effect of Multi-Species Probiotic Supplementation on Fecal Microbiota in Pre-Weaned Holstein Dairy Calves in California

**DOI:** 10.3390/microorganisms13081810

**Published:** 2025-08-02

**Authors:** Yoonsuk Lee, Heidi A. Rossow, Deniece R. Williams, Sejin Cheong, Hedmon Okella, Logan Widmer, Emmanuel Okello

**Affiliations:** 1 Veterinary Medicine Teaching and Research Center, School of Veterinary Medicine, University of California Davis, Tulare, CA 93274, USA; yoonsuk.lee@wisc.edu (Y.L.);; 2 Department of Population Health & Reproduction, School of Veterinary Medicine, University of California Davis, Davis, CA 95616, USA

**Keywords:** diarrhea, probiotic, gut microbiota, pre-wean dairy calves, randomized trial

## Abstract

The gross benefit of feeding multi-species probiotics has been reported, but the effect on the gut microbiota in pre-weaned dairy calves has not been elucidated. To address this gap, a randomized controlled trial was conducted in California, USA, to investigate the effect of feeding probiotics on the fecal microbiota of pre-weaned dairy calves. A total of 30 neonatal calves were randomly assigned to either the probiotic (PRO) or control (CON) treatment. Fecal samples were collected at four age timepoints: days 7, 14, 21, and 42. Fecal bacterial population was analyzed using 16S rRNA amplicon sequencing. Differential abundance analysis was conducted to investigate the difference between the PRO and CON treatments, and diarrheic and non-diarrheic calves in each PRO and CON group. The PRO group had decreased *Clostridium perfringens* and *Fusobacterium varium* compared to the CON at 7 days of age. At 7 days of age, diarrheic calves in CON had more abundant *F. varium* compared to non-diarrheic calves, but there was no difference between diarrheic and non-diarrheic calves in the PRO group. In conclusion, probiotics administration decreased the population of pathogenic bacteria in feces from pre-weaned dairy calves on Day 7 of age. However, the treatment did not have an impact on bacterial diversity. These results suggest that the administration of probiotics has the potential to control gastrointestinal pathogens.

## 1. Introduction

Diarrhea is one of the most common diseases in dairy calves and accounts for 56.4% of pre-wean calf deaths in the United States [1]. Calfhood diseases significantly affect mortality and growth performance and are linked to decreased milk yield in the first lactation [2,3]. Therefore, preventing diarrhea plays a key role in ensuring sustainable dairy farming from both animal welfare and economic perspectives.

Antimicrobial drugs (AMDs) have traditionally been the primary option for treating calf diarrhea. However, there is increasing concern about antimicrobial resistance (AMR), which reduces the effectiveness of these treatments and poses a potential risk to human health [4,5]. In California, for example, a recent study found that 50.8% of Salmonella isolates from cattle displayed multidrug resistance [6]. To help reduce the use of antimicrobials (AMDs), researchers have proposed feeding with probiotics as a viable alternative treatment option [7].

The World Health Organization defines probiotics as live microorganisms that, when administered in adequate amounts, provide health benefits to the host [8]. Probiotics enhance gut health by preventing pathogenic bacteria from colonizing the intestines and modulating gut mucosal immunity [9]. Different probiotic bacteria strains offer various beneficial effects. *Bacillus subtilis* and *Bacillus lichenformis* have been shown to enhance the growth rate, boost immune function, and reduce fecal pathogen shedding [10,11,12]. *Lactobacillus animalis* and *Propionibacterium freudenreichii* have been reported to increase milk yield by improving feed efficiency and stabilizing rumen pH [13]. It is reported that feeding a combination of *Lactobacillus* strains reduced the incidence and average duration of diarrhea while increasing average daily gain (ADG) in pre-wean Holstein calves [14].

While there is documented evidence of the benefits of feeding with probiotics, the effectiveness of probiotics in altering fecal microbiota is not fully understood [15]. Altered microbiota can serve as an indicator of improved metabolic health and physiological benefits [16,17]. Furthermore, the implications of administering multi-strain probiotics on fecal microbiota at various ages in calves, both with and without diarrhea, remain insufficiently characterized. Consequently, we aimed to characterize the effect of feeding multi-species probiotics on the composition and dynamics of the fecal bacterial population in pre-weaned dairy calves using 16S sequencing.

The application of 16S sequencing has been widely used to investigate bacterial composition across various sample types [18]. This technique targets specific ribosomal genes in bacteria and assists in classifying bacteria at the genus or species taxonomic level, making it one of the most time-efficient and cost-effective methods [19]. Since 16S amplicon sequencing targets only a small portion of the bacterial genome, this technique is limited in its ability to resolve bacterial diversity in depth [20].

In this pilot study, we analyzed the changes in the fecal bacterial population of calves by measuring alpha and beta diversity between the probiotic and placebo treatments at days 7, 14, 21, and 42. Additionally, we compared the relative abundance of bacterial populations between the probiotic and placebo treatments and evaluated the effect of diarrhea events within each treatment group using differential abundance analysis.

## 2. Materials and Methods

All procedures were approved by the University of California, Davis, Institutional Animal Care and Use Committee (Protocol #: 22245).

### 2.1. Study Design

This study was conducted on a 5500-lactating-cow dairy herd located in Kings County, California, as part of a larger field trial to evaluate the effectiveness of probiotics on weight gain and rumen development. The study design is described using the CONSORT diagram (Figure S1). Briefly, neonatal Holstein heifer calves (*n* = 329) were enrolled in a block-randomized field trial between 4 October and 6 December 2021. The sample size for the primary study was determined to identify an average daily gain of 900 g/day with a standard deviation of 31 g [12]. Calves were blocked by hutch row and were randomly assigned to the treatment groups using a random number generator in Microsoft Excel (Microsoft Corporation, Redmond, WA, USA). Treatment groups were as follows: (1) placebo treatment (CON) comprising 176 calves, which were administered 0.5 g of lactose (Sigma-Aldrich Corp, St Louis, MO, USA) in milk once per day from birth to weaning at 60 days and (2) probiotic treatment (PRO), comprising 153 calves which were administered 0.5 g (1.1 × 10^10^ CFU^2^/g) of probiotic product (Bovamine Dairy Plus, Chr. Hansen, Milwaukee, WI, USA) in milk once per day from birth to weaning at 60 days. The probiotic product consisted of *Bacillus subtilis* (4.4 × 10^9^ CFU/g), *Bacillus lichenformis* (4.4 × 10^9^ CFU/g), *Lactobacillus animalis* (1 × 10^9^ CFU/g), and *Propionibacterium freudenreichii* (2 × 10^9^ CFU/g). The lactose powder was given to CON to match the lactose content in the probiotic treatment solution for PRO.

The farm staff examined all the calves prior to enrollment, and any calves with gross congenital defects were excluded from the study. Blood samples were collected between 24 and 48 h after birth to measure serum total protein (STP). Calves with STP < 5.1 g/dL were considered to have poor passive transfer of antibodies [21] and excluded from the study.

Among the calves enrolled in the primary study, 30 calves (PRO = 15 calves, CON = 15 calves) were randomly selected for microbiota analysis using a random number generator from Microsoft Excel software (Microsoft Corporation, Redmond, WA, USA). The sample size was determined based on a previous study that showed beneficial effects of feeding probiotics on health outcomes [22].

### 2.2. Calf Management and Administration of Probiotic

Farm staff oversaw the daily management of the calves. They monitored daily calf health and administered treatments based on farm protocols. The antimicrobial drug (AMD) treatment records were retrieved from DairyComp 305 (Valley Agricultural Software, Tulare, CA, USA) by the study staff.

In the maternity barn, calves were fed 4 L of colostrum within 30 min after birth and an additional 2 L 10 h later. Calves were then moved to individual hutches in the calf-rearing area within 24 h of birth. Individual metal wire calf hutches spaced one meter apart were bedded with almond shells and rice hulls. After moving to the rearing area, calves were fed twice daily (at 06.00 h and 13.00 h) with 3 L of milk replacer (Plasma 26:20 P, American Calf Products, Turlock, CA, USA). Both the PRO and CON groups received ad libitum access to starter grain beginning on Day 1 of age. The nutrient composition of the starter grain is detailed in Table S1.

Probiotic and lactose solutions were dissolved with 5 mL of deionized water and administered to either PRO or CON calves, respectively. Solutions were added directly to each respective bottle just before presenting the bottle to the calves.

### 2.3. Fecal Sample Collection, Sample Storage, and Fecal Scoring

Fecal samples were collected from each calf at four timepoints: 7, 14, 21, and 42 days of age. Fecal samples were scored after collection using the scoring system from a previous study [23]. In brief, fecal samples were visually observed and scored on a scale from 1 to 3, with 1 being solid, 2 being pasty, and 3 being watery. Scores of 2 and 3 were considered diarrhea.

To collect fecal samples, the perianal skin was cleaned using gauze soaked in 70% isopropyl alcohol prior to sample collection. Fecal samples were collected from each calf by inserting a gloved and lubricated finger into the rectum. A fresh glove was used for each calf to ensure hygiene and prevent cross-contamination. Fecal samples were transferred from the glove to 50 mL sterile tubes, immediately stored on wet ice, and transported to UC Davis Veterinary Medicine Teaching and Research Center (Tulare, CA, USA) for processing. One g of each sample was aliquoted into a 1.5 mL sterile centrifuge tube and stored at −80 °C.

### 2.4. DNA Extraction, 16S rRNA Sequencing, and Data Analysis

After thawing frozen samples at room temperature for 10 min, DNA extraction was performed using QIAgen PowerFecal Pro DNA Kit (QIAgen, Hilden, Germany, catalog number 51,804) following the manufacturer’s instructions. DNA quantity and quality were evaluated using NanoDrop spectrophotometer (Thermo Fisher Scientific, Wilmington, DE, USA) and samples were submitted to the UC Davis Genome Center for sequencing according to the Center’s protocols.

Amplification of the V3–V4 domain of the 16S rRNA was performed in a two-step PCR procedure using 341F and 806R primers (IDT, San Diego, CA, USA). In step one, both forward and reverse primers contained an Illumina tag sequence, a variable-length spacer to increase diversity and improve the quality of the sequencing run, a linker sequence, and the 16S target sequence. In step two, each sample was barcoded with a unique forward and reverse barcode combination using forward primers with an Illumina P5 adapter sequence, a unique 8 nt barcode, a partial matching sequence of the forward adapter used in step one, and reverse primers with an Illumina P7 adapter sequence, unique 8 nt barcode, and a partial matching sequence of the reverse adapter used in step one.

The final product was quantified on the Qubit instrument using the Qubit High Sensitivity dsDNA kit (Invitrogen, Carlsbad, CA, USA), and individual amplicons were pooled in equal concentrations. The pooled library was cleaned utilizing Ampure XP beads (Beckman Coulter, Brea, CA, USA), then checked for quality and proper amplicon size on an Agilent 2100 Bioanalyzer (Agilent Technologies, Santa Clara, CA, USA). The library was quantified via qPCR followed by 300 bp paired-end sequencing using an Illumina MiSeq instrument (Illumina, San Diego, CA, USA) in the Genome Center DNA Technologies Core (University of California, Davis, CA, USA). Library preparation was performed by the UC Davis Host Microbe Systems Biology Core Facility. Sequenced FASTQ files were processed with a pipeline workflow in QIIME2-2023.5 with DADA2 (version 2) for filtering and trimming sequences [24]. Forward trim length of 265 and reverse trim length of 185 were used to achieve the largest number of reads. Processed amplicons were annotated with SILVA (version 138.2) 138 ribosomal RNA database with a similarity threshold of 99%. The final amplicon sequence variants (ASV) were extracted from QIIME2 and converted into the R program, using the “Qiime2R” package (version 0.99.20) [25].

### 2.5. Statistical Analysis

The prevalence of diarrhea between the treatment groups (i.e., PRO and CON) in each age group was summarized and compared using Fisher’s exact test.

To assess the abundance of taxa in each sample, alpha diversity was measured using the Shannon index and Faith’s phylogenetic diversity (PD) index, which accounts for the total branch length in the phylogenetic tree. To compare the alpha diversities among different age groups within each treatment, the Wilcoxon signed-rank test was used because the fecal samples were collected from the same individuals over time.

For beta diversity, weighted Unifrac was used to evaluate the dissimilarity between treatment groups and among age groups, and a non-metric multidimensional (NMDS) plot was generated to visualize the distance among samples. PERMANOVA 0.2.0 was analyzed using the Adonis2 (16.2) function from the “vegan” package [26] and was used to test the difference in clusters with animal ID as a random effect.

Differential abundance analysis (DAA) was conducted using the DESeq2 package [27]. All statistical analyses were performed using R (4.2.2). A significance level of 0.05 was applied to identify the difference in alpha and beta diversities, while a significance level of 0.01 was used to identify significant bacterial species for DAA.

Power analysis is conducted using two sample *t*-test function in “pwr” package in R [28]. The Shannon index was used for the power analysis with a significance of 0.05.

## 3. Results

### 3.1. Descriptive Data, Sequencing Outcomes, and Power Analysis

A total of 30 calves were included in the bacterial population analysis. All 120 extracted DNA samples (2 treatment groups × 15 calves × 4 sampling timepoints) satisfied the quality control (Quantity of nucleic acid > 20 ng/μL, A260/280 > 1.8) and were submitted for sequencing.

**Figure 1:**
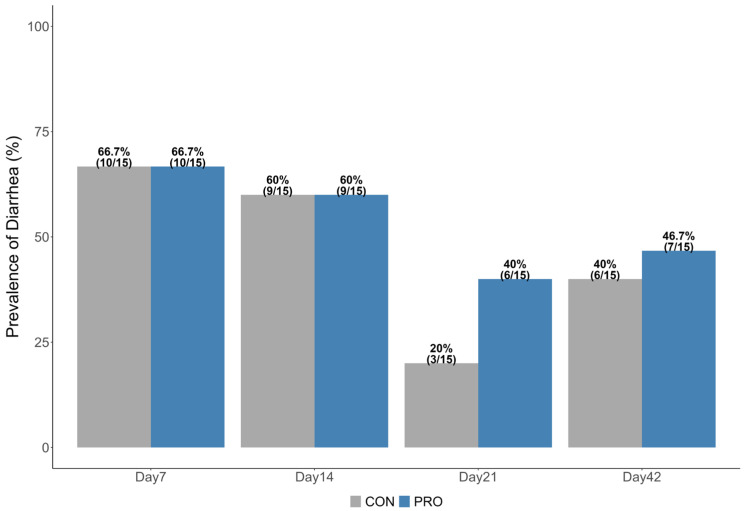
Prevalence (%) of diarrhea at sample collection over each age. The fecal samples were scored using a 3-point scoring system (1 = solid, 2 = pasty, 3 = watery). Prevalence was calculated as (The number of diarrheic samples)/(Total number of collected samples in each treatment group at the timepoint). Scores of 2 and 3 were considered diarrhea. The difference in diarrhea prevalence between two treatment groups was analyzed using Fisher’s exact test, and there was no difference in each age.

The prevalence of diarrhea at sampling is shown in Figure 1. No significant difference in diarrhea prevalence was observed between the PRO and CON groups.

The mean sequencing length per sample was 401.64 bp (Range: 265–430 bp, SD: 42.48 bp), and a total of 2671 ASVs were acquired from 120 samples after the analysis with QIIME2 workflow.

From the power analysis using a two-sample *t*-test with the Shannon diversity index, it was found that the power of this study was 13.7% (α = 0.05).

### 3.2. Relative Abundance

The top five most abundant phyla were revealed from descriptive abundance analysis. The top five most abundant taxa were the same across each sampling time point, but the ranking order of relative abundance by taxa differed depending on the age groups.

**Figure 2:**
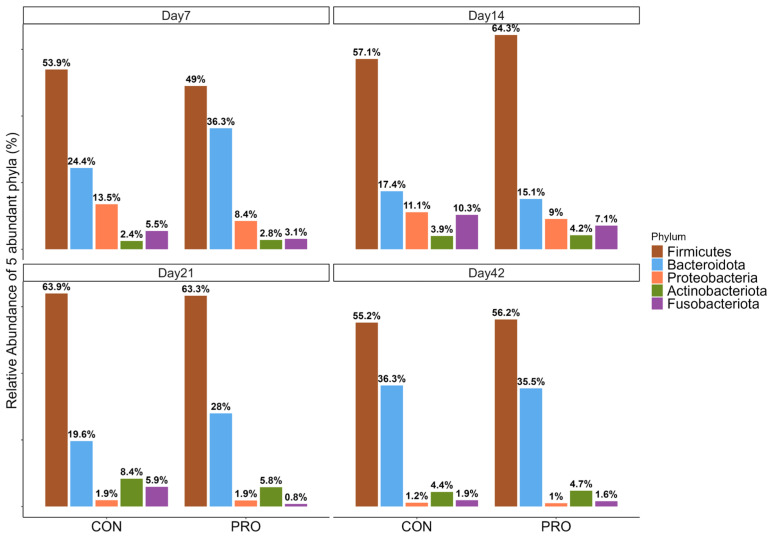
The top five most abundant phylum taxa at each different sampling timepoint (PRO = probiotic treatment group; CON = placebo group).

Overall, Day 7 and Day 14 had the same order of abundant phylum, and Day 21 and Day 42 had the same order of abundant phylum. On Day 7 and Day 14, the top five taxa, ordered from the most to least abundant, were as follows: *Bacillota*, *Bacteroidota*, *Pseudomonadota*, *Fusobacteriota*, and *Actinobacteriota*. On Day 21 and 42, the order of the most to least abundant phylum was as follows: *Bacillota*, *Bacteroidota*, *Actinobacteriota*, *Fusobacteriota*, and *Pseudomonadota* (Figure 2). At all ages, *Fusobacteriota* was more abundant in the CON than PRO group.

The relative abundance of taxa at the genus level between the PRO and CON groups was visualized with a heatmap to provide an overview of the different patterns (Figure S2).

### 3.3. Alpha and Beta Diversity

**Figure 3:**
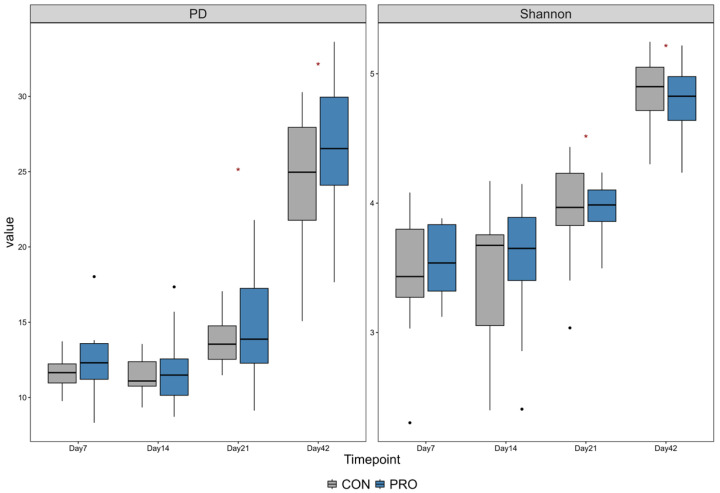
Quantile box plot of Shannon and Phylogenetic diversity (PD) index between two treatment groups at each age. Gray = CON; blue = PRO. Wilcoxon signed rank test was used to determine the statistical difference for each comparison between age groups. Significance is marked with red asterisk (*p* < 0.05).

Day 42 had the highest PD index and Shannon index among all ages. In both PRO and CON, the Shannon and PD indices on Day 7 and 14 were not different. However, the PD indices from Day 7 and 21 were not different in the PRO group but were higher on Day 21 than Day 7 and 14 in the CON group (*p* < 0.001). In contrast, the Shannon indices differed between Day 7 and Day 21 for both PRO and CON. Differences were found in the following comparisons: Day 7 vs. 42, Day 14 vs. 21, Day 14 vs. 42, and Day 21 vs. 42. The Shannon and PD indices increased as calves aged (Figure 3).

**Figure 4:**
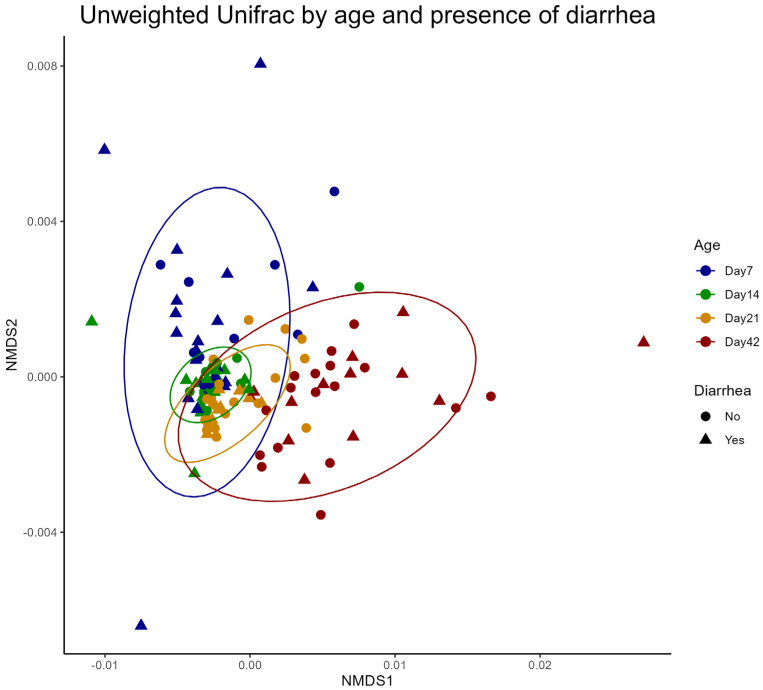
Weighted Unifrac NMDS plot comparing each sampling timepoint and the presence of diarrhea. No difference was observed between PRO and CON at each age. PERMANOVA test was used to detect statistical significance: Comparison between PRO and CON in Day 7 (*p* = 0.853). Comparison between PRO and CON in Day 14 (*p* = 0.809). Comparison between PRO and CON in Day 21 (*p* = 0.549). Comparison between PRO and CON on Day 42 (*p* = 0.615). The ellipse represents 95% confidence level for a multivariate t-distribution.

The beta diversity calculated using weighted Unifrac was assessed in relation to age and diarrhea status (Figure 4). There was no difference in beta diversity between the PRO and CON groups at each age stage.

### 3.4. Differential Abundance Analysis

**Figure 5:**
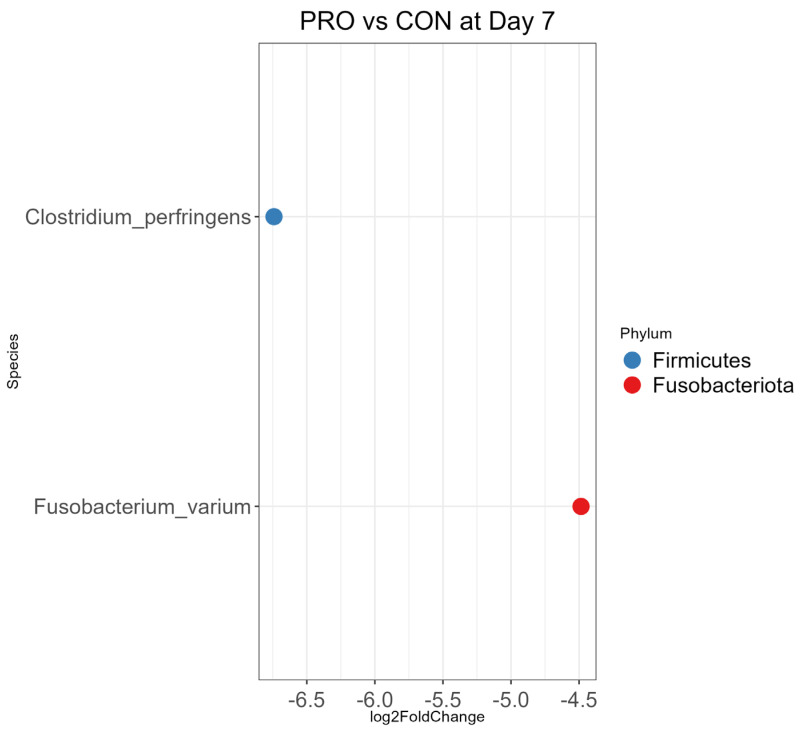
Bacterial species differential abundance analysis comparing probiotic treatment (PRO) and placebo (CON) groups at Day 7. The log2-fold change was measured for the differential abundance between PRO vs. CON with CON as a reference. Only statistically significant species are included in the plot (*p* < 0.01), and the color is indicated by each node’s phylum.

On Day 7, *Clostridium perfringens* (0.01 times abundance) and *Fusobacterium varium* (0.05 times abundance) were less abundant in the PRO group than the CON (*p* < 0.01, Figure 5). The abundance pattern was different on Day 42 compared to Day 21 (Figure S3B) for *Lactobacillus amylovorus* which was more abundant in the PRO group than the CON (*p* < 0.01, Figure S3C).

**Figure 6:**
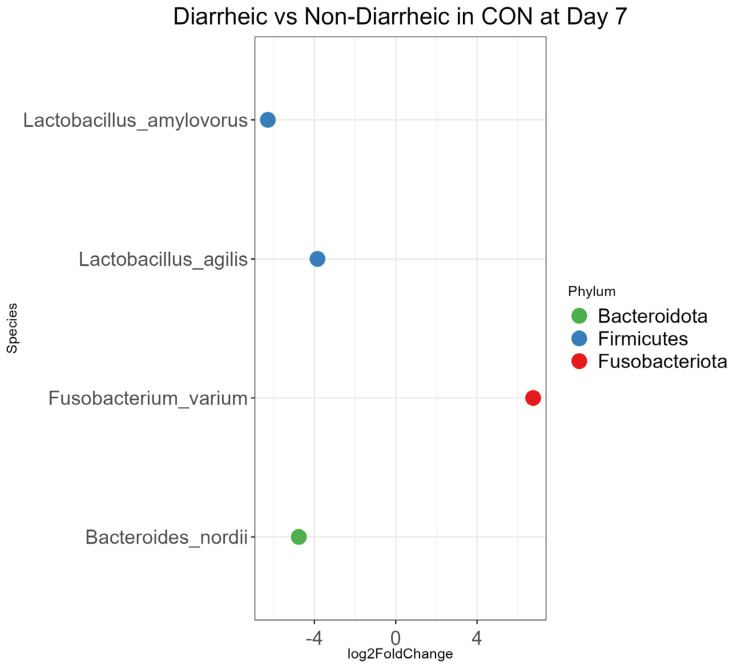
Bacterial species differential abundance analysis comparing diarrheic and non-diarrheic calves in placebo (CON) group. Log2-fold change was measured for the different species abundance between diarrheic vs. non-diarrheic calves, with non-diarrheic calves as reference. Only statistically significant species are included in the plot (*p* < 0.01), and the color is indicated by each node’s phylum.

The DAA between diarrheic and non-diarrheic calves within the PRO and CON groups at each age are described below (Figure 6 and Figure S5). On Day 7, *Fusobacterium varium* was 107.60 times more abundant in CON diarrheic calves than CON non-diarrheic calves (*p* < 0.01, Figure 6). On Day 14, *Lactobacillus amylovorus* was the only species that was different between CON diarrheic calves and CON non-diarrheic calves. *Lactobacillus amylovorus* was 100.00 times more abundant in CON non-diarrheic calves compared to CON diarrheic calves (*p* < 0.01, Figure S5C). On Day 21, PRO diarrheic calves had more abundant *Lactobacillus mucosae*, *Lactobacillus amylovorus*, and *Lactobacillus reuteri* compared to PRO non-diarrheic calves. Also, *Clostridium* sp. was 16 times more abundant in CON diarrheic calves compared to CON non-diarrheic calves (*p* < 0.01, Figure S5D,E). On Day 42, *Eubacterium coprostanoligenes* had 0.003 times the abundance in the PRO diarrheic calves compared to the PRO non-diarrheic calves. However, *Eubacterium coprostanoligenes* was 24.25 times more abundant in CON diarrheic calves compared to CON non-diarrheic calves (*p* < 0.01, Figure S5F,G).

## 4. Discussion

### 4.1. Diarrhea Prevalence, Relative Abundance, and Power Analysis

We did not observe a statistical difference in the prevalence of diarrhea between the group fed with probiotics (PRO) and the group that did not receive probiotics (CON). This could be due to the limited sample size, as the study was not designed to specifically identify the difference in the prevalence of diarrhea. This finding is consistent with previous studies that did not find an effect of feeding with probiotics on diarrhea, particularly in calves without nutritional stress [29,30]. However, another study reported that feeding with *Bacillus subtilis* decreased the diarrhea rate [31].

On Day 7, the CON group had numerically more abundant *Pseudomonadota* and Fusobacteriota (Figure 2). A previous study found that diarrheic calves had more abundant *Pseudomonadota* compared to non-diarrheic calves [32], and increased *Fusobacteriota* is often related to dysbiosis [33]. From Day 7 to 14 (Figure 2), we found that the order of the top five most abundant bacterial phyla was the same. However, the pattern changed after Day 21 (Figure 2), which indicates a dynamic shift in the calf bacterial population during the pre-wean period. This finding is consistent with a previous study where they found the order of abundant bacterial phyla shifted between week 3 and week 4 in neonatal dairy calves [34]. However, they found that *Pseudomonadota* was the most increased bacterial phylum, whereas this study found that Bacteroidetes was the most increased phylum between week 3 and week 4. It is possible that this was due to the environmental differences in the farm management.

The power of this study was 13.7% due to the difference in the Shannon index between the two treatment groups. This indicates that the sample size was far too small to identify the difference in Shannon index, which had little difference with the presence of probiotic treatment. With the Shannon indexes measured for two treatment groups (PRO: Mean 3.55, SD 0.28, CON: Mean 3.47. SD 0.45), the required sample size for a power of 80% to detect the difference in the Shannon index should have been a total of 1098 calves, which was deemed impractical for a field trial. With this small power of the probiotic treatment, in terms of common practice, it appears that the probiotic treatment had a minimal effect in manipulating the overall bacterial diversity in the gastrointestinal system in the pre-weaned dairy calves.

### 4.2. Alpha and Beta Diversity

In this study, alpha diversity increased with calf age, and the highest alpha diversity was found at Day 42 of age. A study showed that alpha diversity indices, including Shannon diversity, increased as calves aged from week 1 to week 4 [35]. However, we could not find a difference in alpha diversity between the PRO and CON groups. A previous study discussed that alpha diversity decreases in diarrheic calves due to competitive exclusion by pathogens limiting microbial diversity [33]. We may not have found a difference in alpha diversity between the PRO and CON groups because the prevalence of diarrhea at each age was not different. In this study, we applied the phylogenetic diversity (PD) index to account for the evolutionary relatedness of bacterial taxa in fecal microbiota in addition to the Shannon index. To our knowledge, this is the first study to apply the PD index to alpha diversity in the fecal microbiota of pre-wean dairy calves. Previous studies used the PD index to evaluate alpha diversity in the lower gut [36] and rumen [37] in pre-weaning calves.

In this study, probiotic treatment did not alter beta diversity. In contrast, previous study found that feeding neonatal Holstein calves multi-species probiotics, including *B. subtilis*, had a dose-dependent effect. Calves with a triple dose of probiotic treatment showed a marked shift in the principal component in the Bray–Curtis distance principal coordinates analysis compared to control and regular dose [31]. This raises speculation that the dose of probiotic treatment in this study might not be high enough to alter the beta diversity. However, the other study found that probiotic feeding was beneficial for decreasing the diarrhea rate, even though the beta diversity was not different between the treatment and non-treatment groups [38]. In this study, the Non-metric Multidimensional scaling (NMDS) plot showed that beta diversity in fecal bacterial populations from calves shifted as calves aged (Figure 4). In the NMDS plot, interindividual dissimilarity decreased as calves aged but increased again on Day 42. This finding is consistent with previous study, which found that interindividual dissimilarity from the gastrointestinal tract decreased from day 7 to day 28 but increased on day 49, as indicated by the segregation of bacterial community in canonical analysis of principal coordinates [39]. A previous study also reported that beta diversity decreases as calves age, as indicated by decreased Unifrac dissimilarity [40]. However, we found that the Unifrac dissimilarity increased on Day 42 again, which disagreed with the previous study [40]. This inconsistency may be due to the different management of the dairy farm, which was not investigated in this study.

### 4.3. Differential Abundance Analysis

For differential abundance analysis (DAA), we used the DESeq2 package in R, which uses a negative binomial model to account for the overdispersion of the dataset inferred from zero-inflated data in absolute abundance from the amplicon sequence variant (ASV) table. DESeq2 model accounts for both within-variability and between-variability in sample groups, which increases precision and provides log 2-fold change between comparing objectives to facilitate the interpretation of the outcome [27]. DAA was conducted at genus level to account for the low resolution of the sequencing method (Figures S4A–D and S6A–H), as well as species level to identify the clinical significance of using probiotic (Figure 5 and Figure 6, Figures S3A–C and S5A–G).

PRO versus CON comparisons at each age indicated that PRO decreased the abundance of pathogens in feces. On Day 7, CON had more abundant *Clostridium perfringens* compared to PRO. *Clostridium perfringens* is correlated with diarrhea in pre-wean dairy calves; however, clinical outcome differs depending on the type of toxin *C. perfringens* secretes [41]. We could not identify the toxin type for *C. perfringens* in the feces since 16S sequencing lacked sufficient sequencing resolution to determine the toxin of *C. perfringens*. The CON group had more abundant *Fusobacterium varium* compared to the PRO group. A previous study reported increased *Fusobacterium varium* is correlated with neonatal calves infected with *Cryptosporidium parvum*, a pathogenic protozoon that can cause diarrhea [42]. Another previous study investigated the amount of fecal shedding of *Cryptosporidium parvum* in pre-wean dairy calves and found that calves who were treated with *B. subtilis* shed a significantly lower amount of *C. parvum* compared to control calves at Day 14 of age [12]. Further study is required to investigate the relationship between *Cryptosporidium parvum* and *Fusobacterium varium* in temporal microbiota dynamics.

On Days 7 and 14, we observed that *Fusobacteriota* was more abundant and *Bacteroidota*, *Bacillota*, *Actinobacteriota*, *Desulfobacterota*, and *Campylobacterota* were less abundant in diarrheic calves compared to non-diarrheic calves (Figure 6 and Figure S5A–C). This agrees with a previous study, which found that diarrheic calves had more abundant *Fusobacteriota* and less abundant *Pseudomonadota*, *Bacteroidota*, and Actinobacteria compared to non-diarrheic calves [43]. However, on Days 21 and 42, this overall trend in phylum abundance shifted (Figure S5D–G). We found that *Bacillota*, *Pseudomonadota*, Actinobacteria, *Bacteroidota*, and *Spirochaetota* were more abundant in diarrheic calves compared to non-diarrheic calves. This shift in microbiota may occur along with age as calves undergo forestomach development and experience an increase in voluntary starter feed intake.

A previous study determined that healthy calves have a lower abundance of *Lactobacillus* species compared to diarrheic calves [44]. However, other studies found that *Lactobacillus* species are beneficial in decreasing diarrhea incidence by inhibiting pathogenic bacteria growth [33,45]. In our study, *Lactobacillus* species were more abundant in non-diarrheic calves than diarrheic calves when calves were on Day 14 age (Figure S5B,C), whereas they were more abundant in diarrheic calves than non-diarrheic calves when calves were at Day 21 (Figure S5D,E). This suggests that the abundance pattern of bacterial taxa in diarrheic calves may shift with age.

On Day 42, the abundance pattern of *Eubacterium coprostanoligenes* in diarrheic calves differed between the PRO and CON treatments (Figure S5F,G). In the PRO group, diarrheic calves had a lower abundance of *E. coprostanoligenes* compared to non-diarrheic calves. However, in CON, diarrheic calves had a higher abundance of *E. coprostanoligenes* compared to non-diarrheic calves. According to the other previous study, *E. coprostanoligenes* was more abundant in the rumen microbiome in high-yield lactating adult cattle than in low-yield cattle. Further study is required to investigate the role of *E. coprostanoligenes* in the fecal microbiota of pre-wean dairy calves [46].

We did not observe that probiotic feeding decreased pathogenic bacteria such as *Clostridium perfringens* and *Fusobacterium varium* shedding after Day 7 of age, so we suggest that the first week after birth may represent the most effective window for feeding probiotics to decrease pathogen shedding in feces.

Our study has some limitations. Since we used 16S-rRNA amplicon sequencing, this pilot study has inherent limitations in the resolution and depth of sequencing, which would allow for a better understanding of bacterial speciation. Further study with a metagenomic approach is required to confirm and analyze a broader spectrum of bacterial species as well as the interconnection between *Cryptosporidium* sp. and *Fusobacterium varium*. We used fecal samples to analyze the microbiota to minimize the invasiveness of sampling, but fecal samples may not represent the whole digestive tract microbiota [47]. Due to the financial constraints of this trial, we enrolled a total of 30 calves, but the power analysis indicated that larger sample size (n = 1098) is required to detect the difference in Shannon index between treatment groups. Given that the gastrointestinal microbiome can be affected by various management (e.g., milk replacer diet vs. pasteurized milk diet), larger sample size and more studies in multiple farms with various management style are required to verify the effectiveness of administration of probiotics.

## 5. Conclusions

We found that the administration of multi-species probiotics had a minimal effect on manipulating overall bacterial diversity in pre-weaned Holstein calves. The post hoc power analysis indicated that the statistical power was 13.7%, based on the calculation of change in the Shannon index between the probiotic treatment and control groups. However, according to the differential analysis, feeding with probiotics reduced the fecal abundance of *Fusobacterium varium* and *Clostridium perfringens* in pre-weaned dairy calves at Day 7 of age. *Fusobacterium varium* may be associated with Cryptosporidiosis, one of the main causes of diarrhea in pre-wean dairy calves.

## Data Availability

The original contributions presented in this study are included in the article/Supplementary Material. Further inquiries can be directed to the corresponding author.

## Supplementary Materials

The following supporting information can be downloaded at: https://www.mdpi.com/article/10.3390/microorganisms13081810/s1, Supplementary Figures S1–S6 are provided online; https://doi.org/10.5281/zenodo.15467357; Table S1. Nutrient and chemical composition of the diets.
