# Supplementary material for: Effect of Multi-Species Probiotic Supplementation on Fecal Microbiota in Pre-Weaned Holstein Dairy Calves in California
Source: Microorganisms. 2025 Aug 2;13(8):1810. doi: 10.3390/microorganisms13081810 (PMC12388631; doi:10.3390/microorganisms13081810)
Supplement: Supplementary file 1 [file microorganisms-13-01810-s001.zip › Table S1_Feed nutrient composition.pdf]

Table S1. Nutrient and chemical composition of the diets

Item	n[1]	Mean	SD
Milk replacer[2], % As-Fed	8		
Fat		2.17	0.84
Pro		3.75	0.18
Lac		5.72	0.16
SNF		10.47	0.32
SCC x 1000		287	84
Milk replacer with PHM[3], % As-Fed	15		
Fat		3.62	0.45
Pro		3.92	0.16
Lac		5.64	0.28
SNF		10.54	0.44
SCC x 1000		483	210
Starter pellet[4], % DM	3		
DM, %		85.70	0.24
CP		23.89	0.46
NDF		21.15	0.29
Fat		4.22	0.28
Starch		27.48	0.34
ASH		6.57	0.22
NFC		46.42	0.35
Ca		1.03	0.16
P		0.56	0.04
Na		0.29	0.08
K		1.29	0.06
Textured grain Mix[5], % DM	5		
DM, %		84.83	0.68
CP		17.73	1.32
NDF		27.04	5.59
Fat		3.31	0.52
Starch		37.37	1.71
ASH		3.49	1.15
NFC		51.50	5.59
Ca		0.86	0.05
P		0.44	0.02
Na		0.27	0.07
K		0.80	0.01
TMR[6], % DM	3		
DM, %		57.09	2.16
CP		19.53	0.04
NDF		28.50	1.04
Fat		2.78	0.15
Starch		25.04	1.51

ASH	8.07	0.20
NFC	43.27	1.10
Ca	1.56	0.02
P	0.48	0.01
Na	0.20	0.04

[1]n = samples analyzed
[2]Milk replacer (1 - 21 d)
[3]Pasturized hospital milk (PHM) blend with milk replacer (22 - 60 d)
[4]Starter pellet (1- 52 d)
[5]Textured grain mix (45 -137 d)
[6]TMR (130-180 d)
