# Supplementary figures and images for: Effect of Multi-Species Probiotic Supplementation on Fecal Microbiota in Pre-Weaned Holstein Dairy Calves in California
Source: Microorganisms. 2025 Aug 2;13(8):1810. doi: 10.3390/microorganisms13081810 (PMC12388631; doi:10.3390/microorganisms13081810)

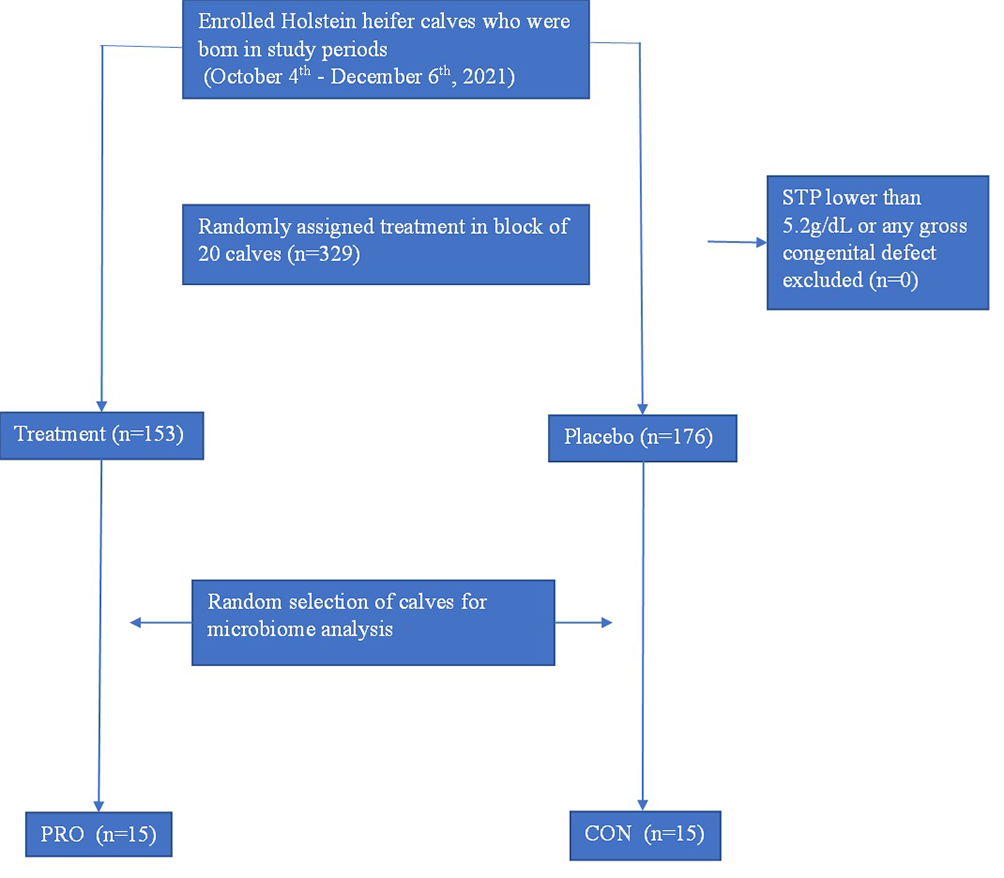

Supplement: Supplementary file 1 [file microorganisms-13-01810-s001.zip › FigS1_CONSORT.tif]

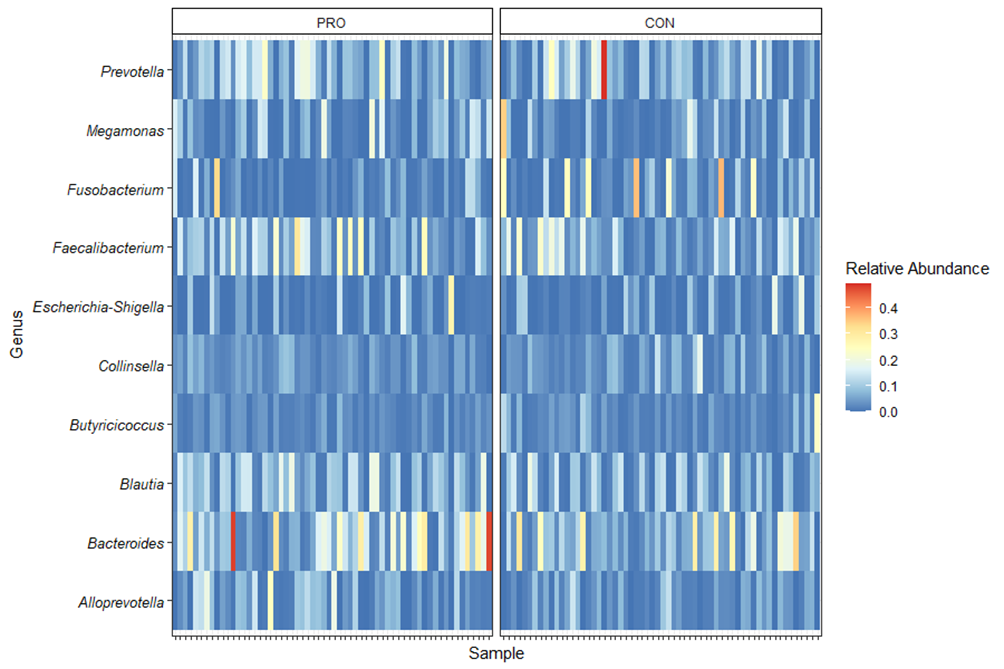

Supplement: Supplementary file 1 [file microorganisms-13-01810-s001.zip › FigS2_Heatmap.tif]

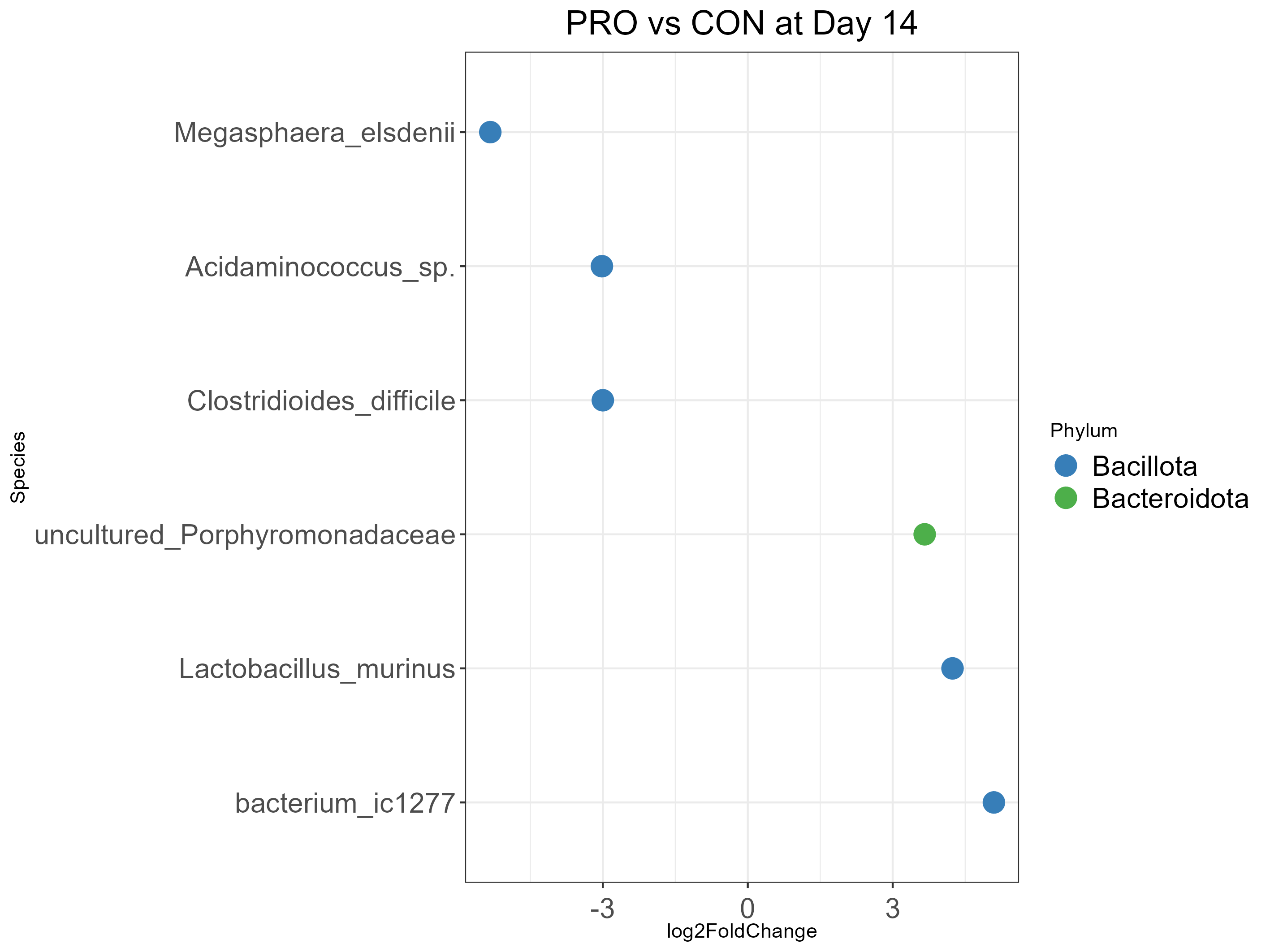

Supplement: Supplementary file 1 [file microorganisms-13-01810-s001.zip › FigS3A_PROvsCON_D14.tiff]

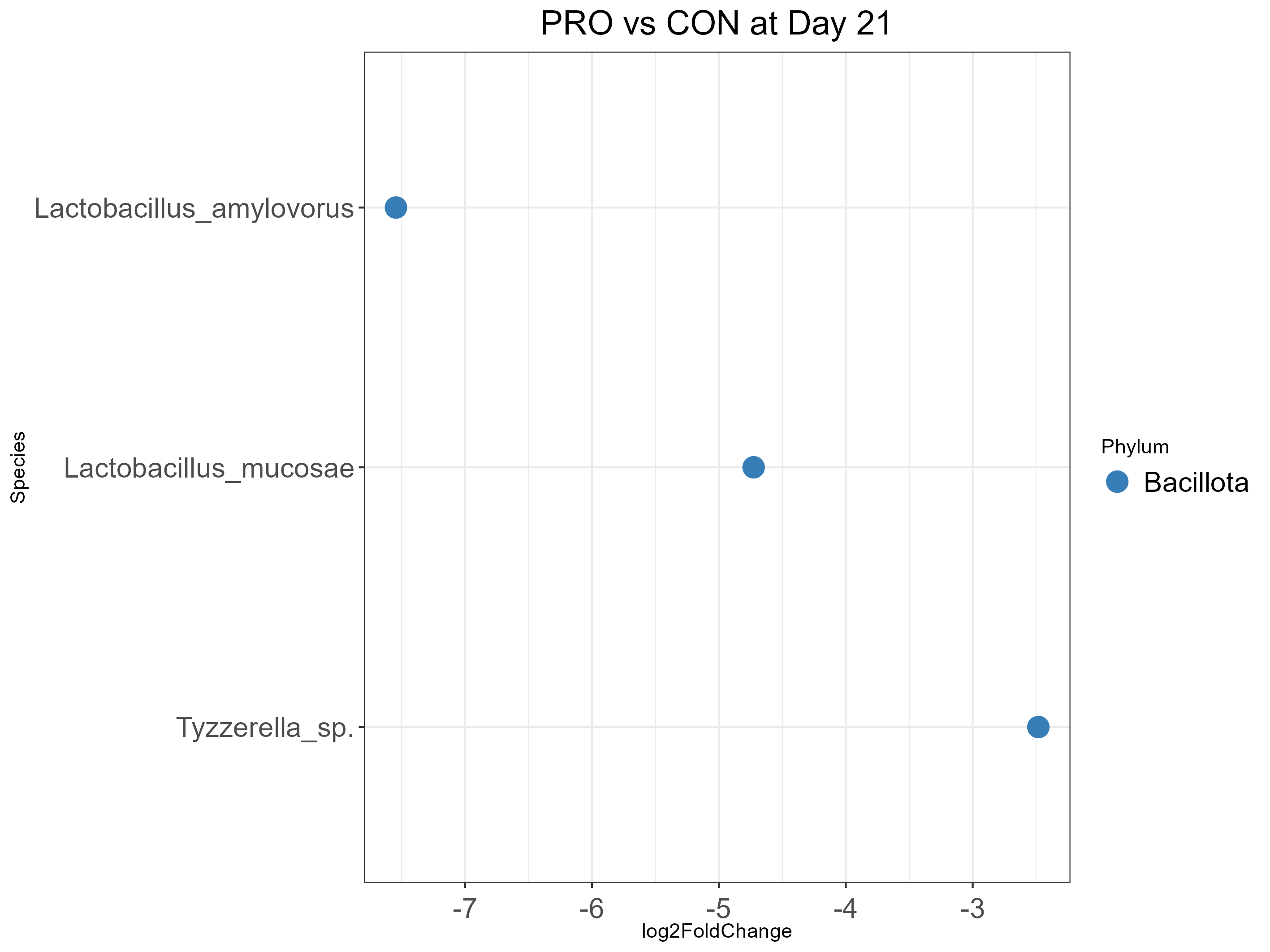

Supplement: Supplementary file 1 [file microorganisms-13-01810-s001.zip › FigS3B_PROvsCON_D21.tiff]

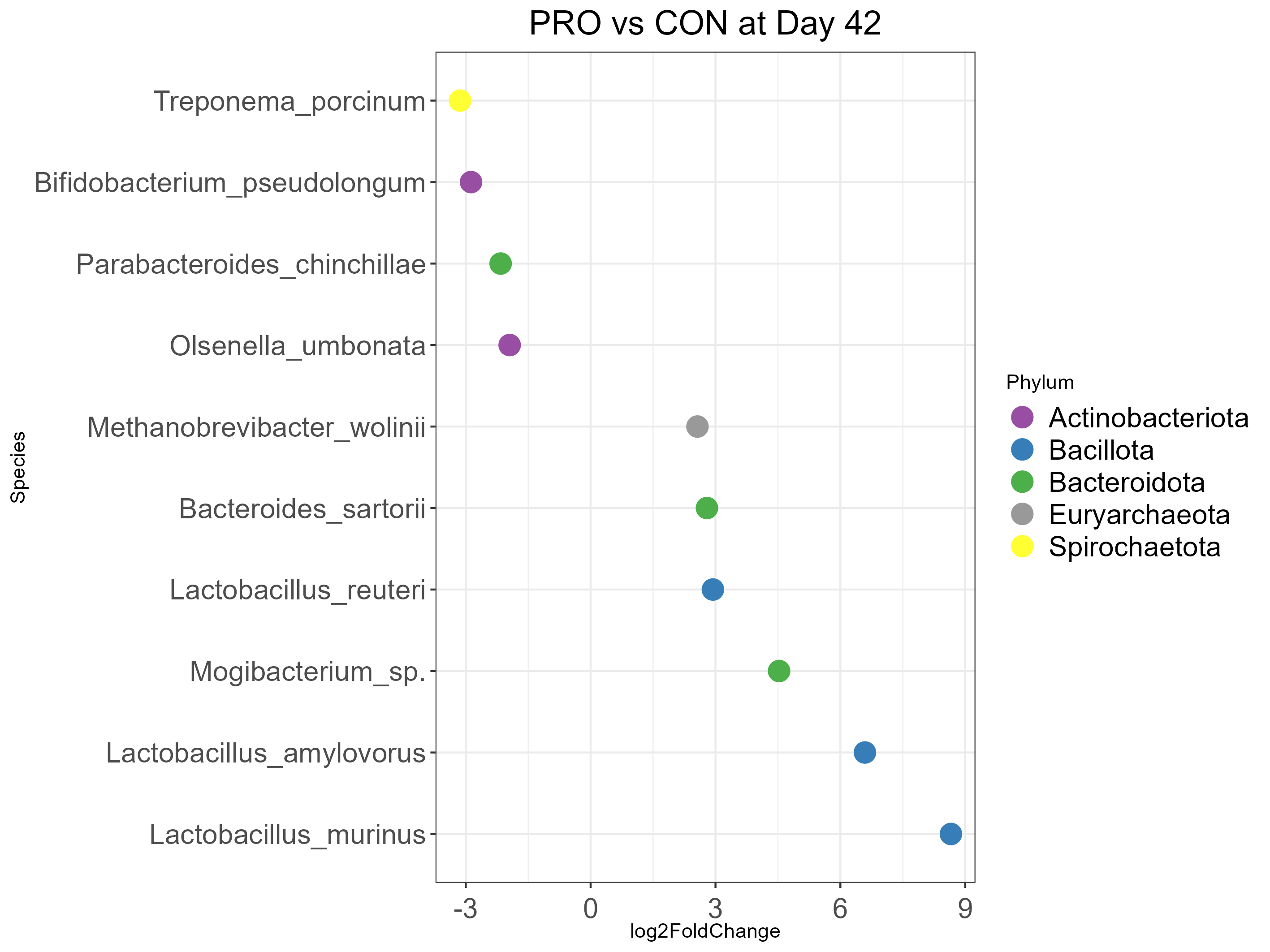

Supplement: Supplementary file 1 [file microorganisms-13-01810-s001.zip › FigS3C_PROvsCON_D42.tiff]

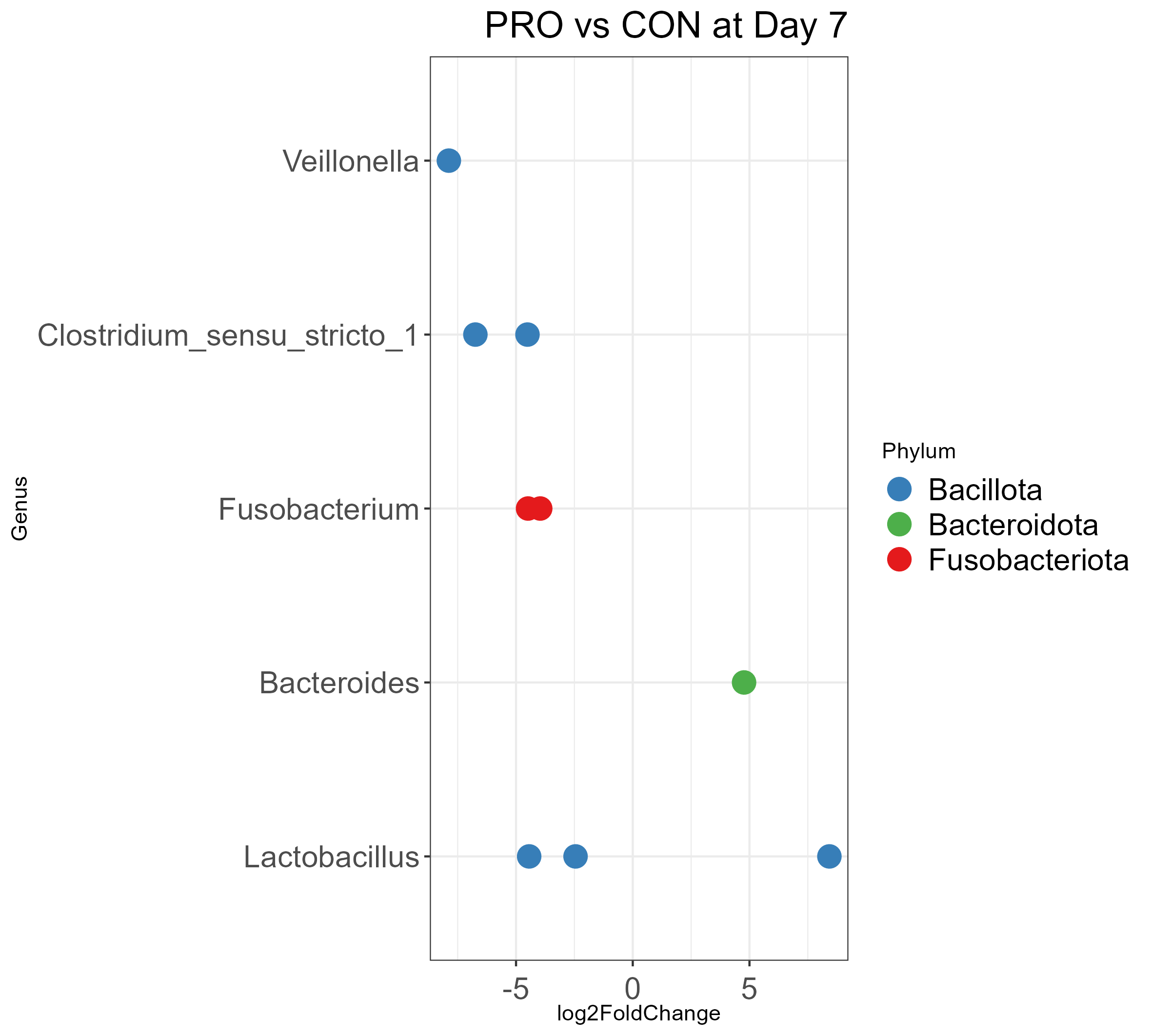

Supplement: Supplementary file 1 [file microorganisms-13-01810-s001.zip › FigS4A_PROvsCON_D7_Genus.tiff]

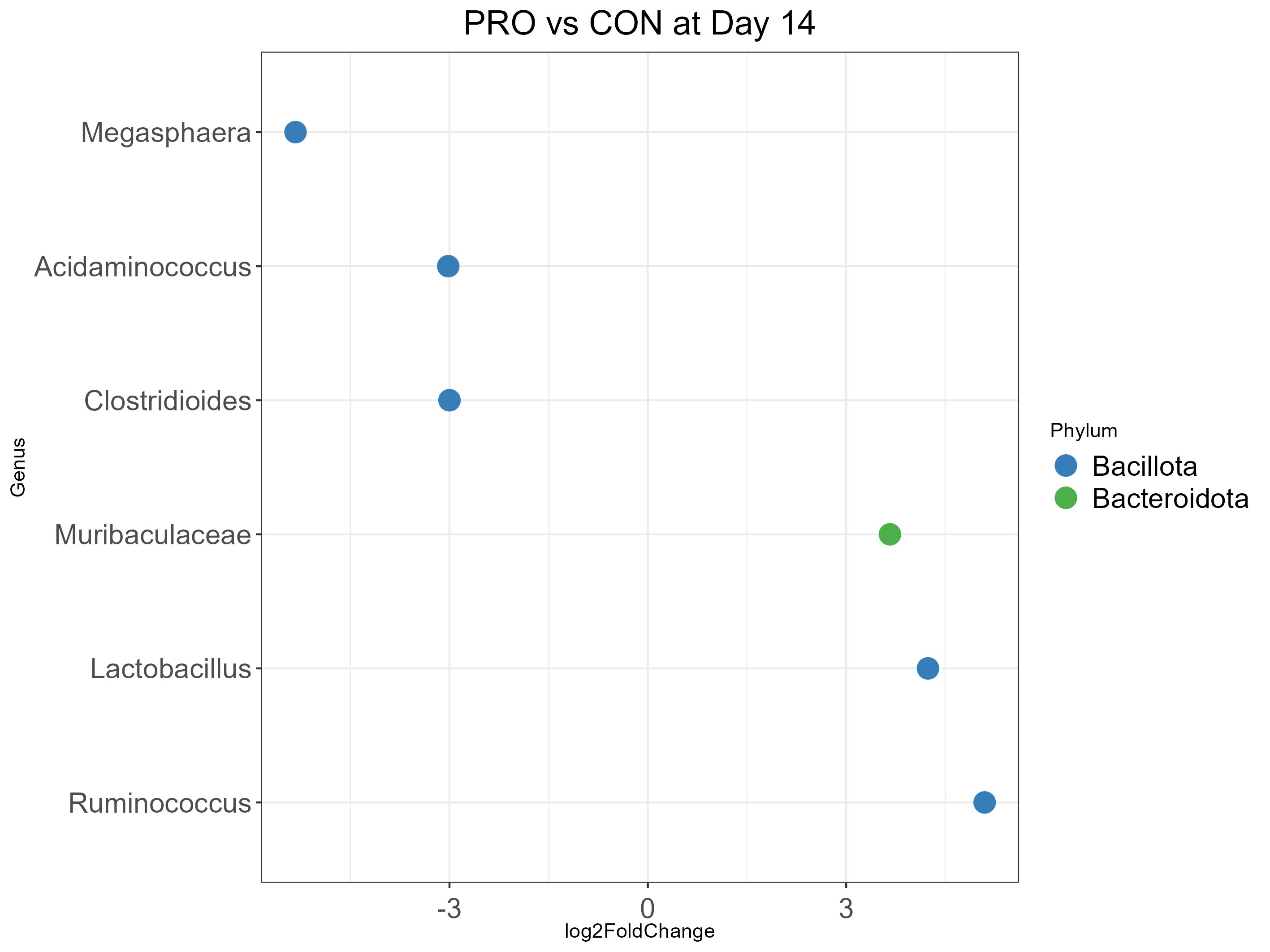

Supplement: Supplementary file 1 [file microorganisms-13-01810-s001.zip › FigS4B_PROvsCON_D14_Genus.tiff]

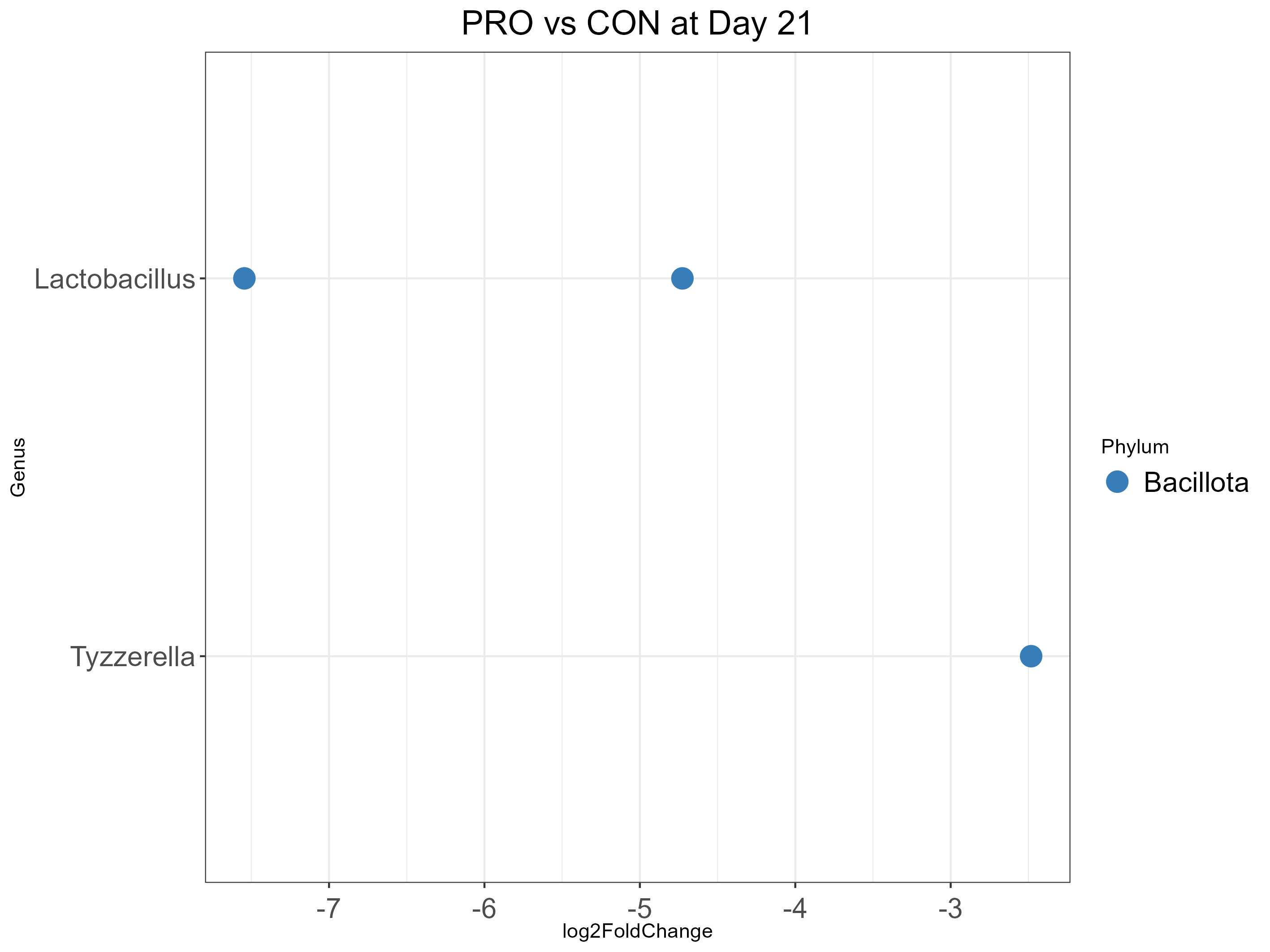

Supplement: Supplementary file 1 [file microorganisms-13-01810-s001.zip › FigS4C_PROvsCON_D21_Genus.tiff]

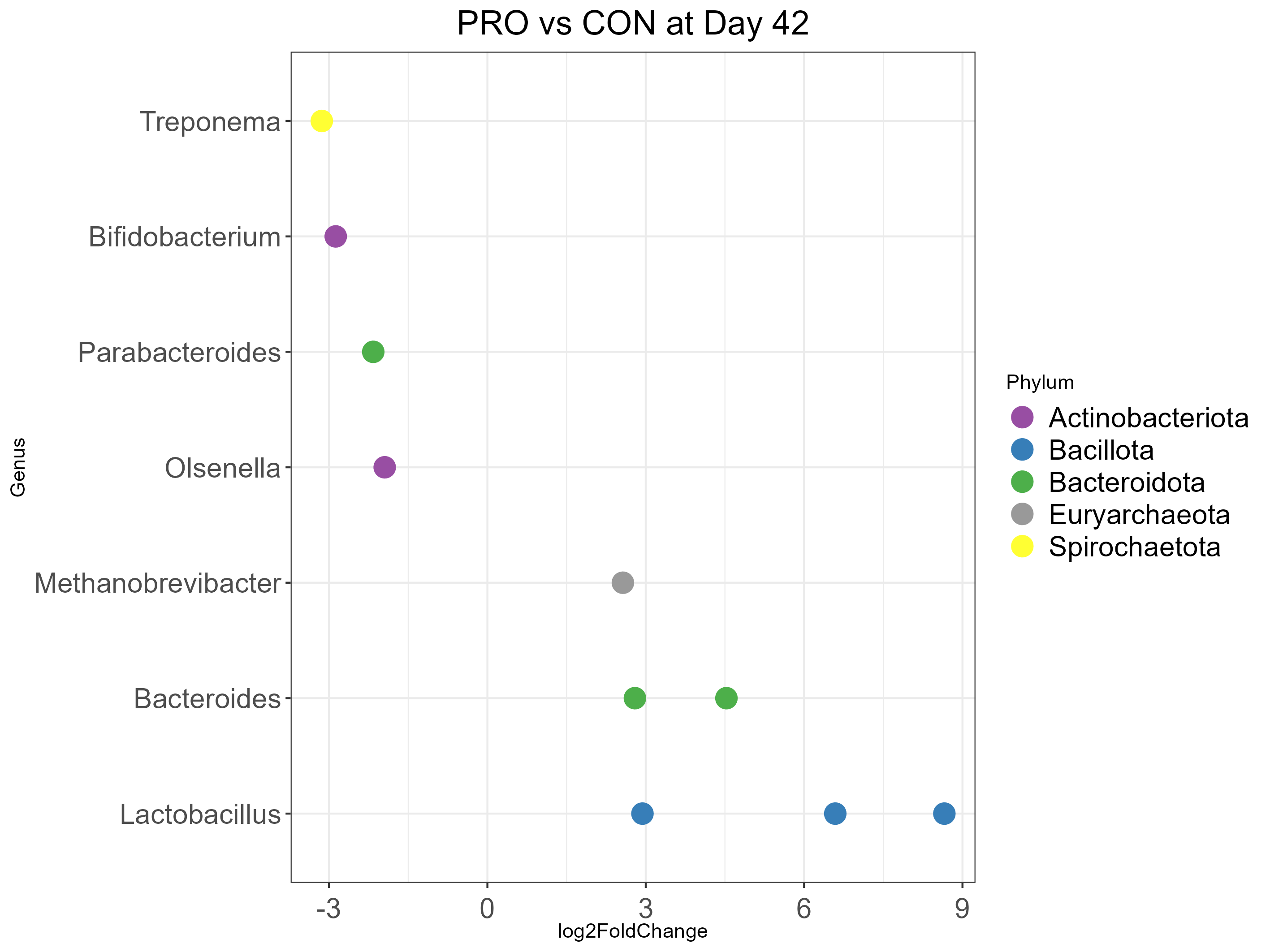

Supplement: Supplementary file 1 [file microorganisms-13-01810-s001.zip › FigS4D_PROvsCON_D42_Genus.tiff]

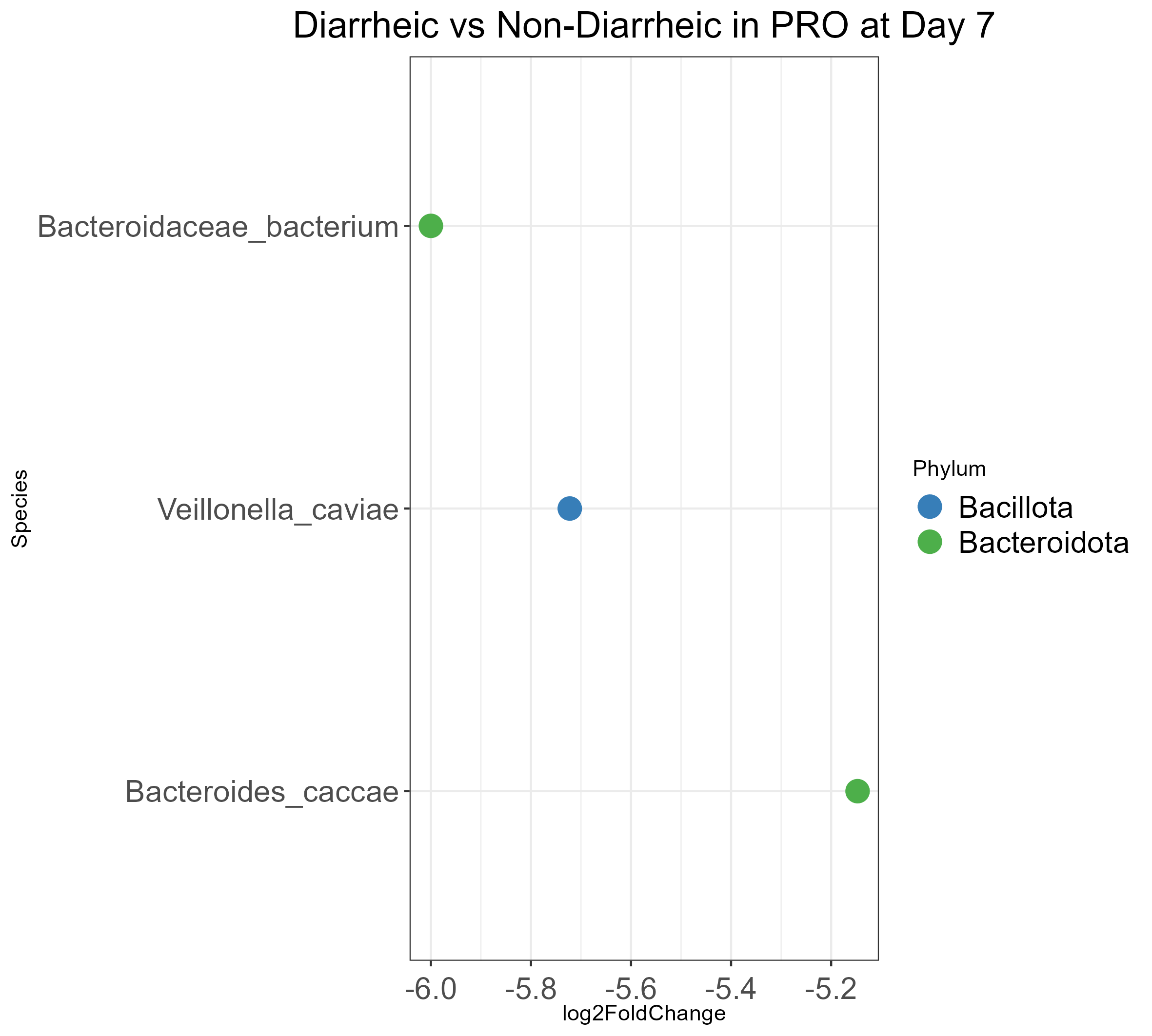

Supplement: Supplementary file 1 [file microorganisms-13-01810-s001.zip › FigS5A_Diarrheic_PRO_D7.tiff]

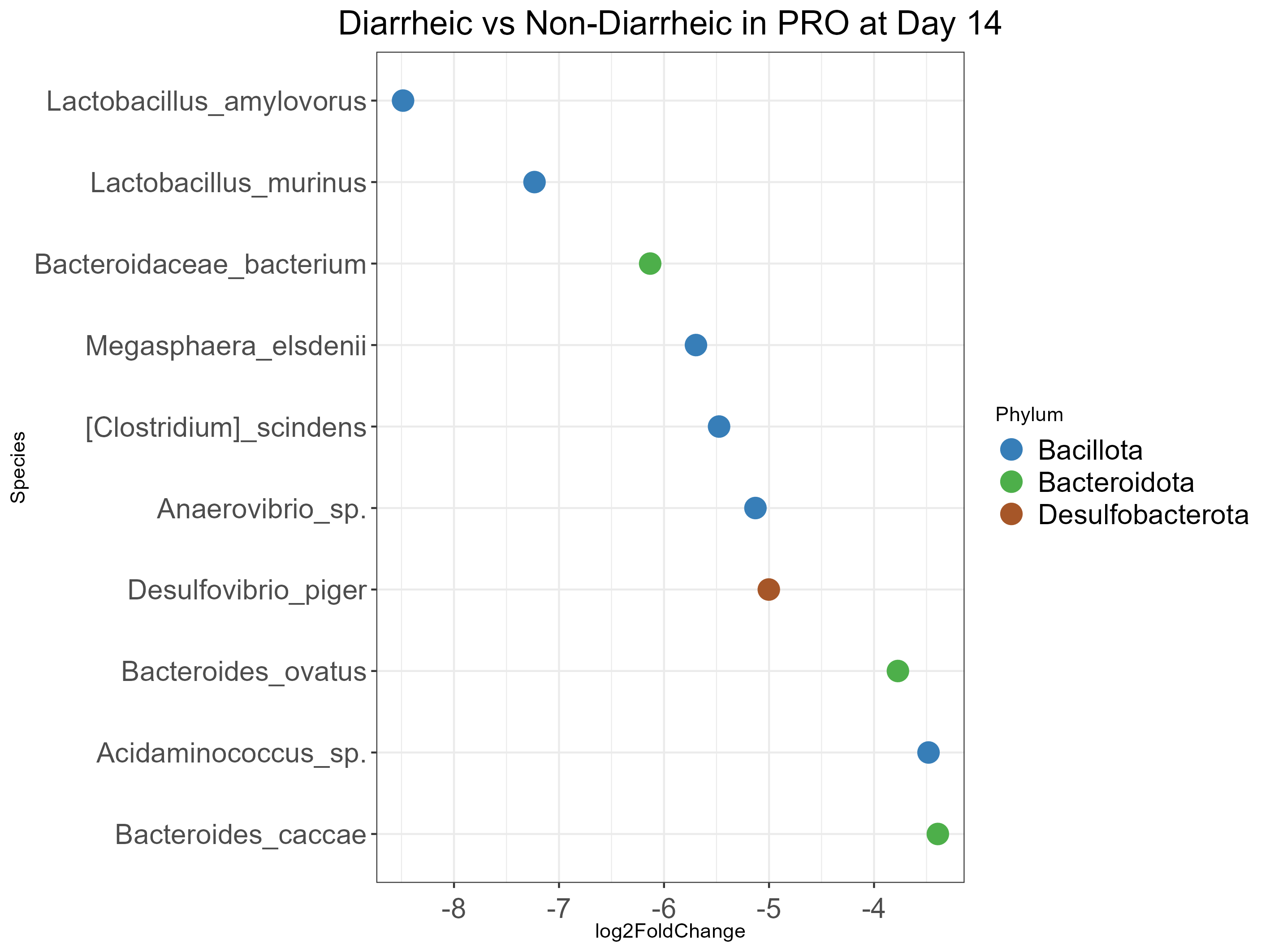

Supplement: Supplementary file 1 [file microorganisms-13-01810-s001.zip › FigS5B_Diarrheic_PRO_D14.tiff]

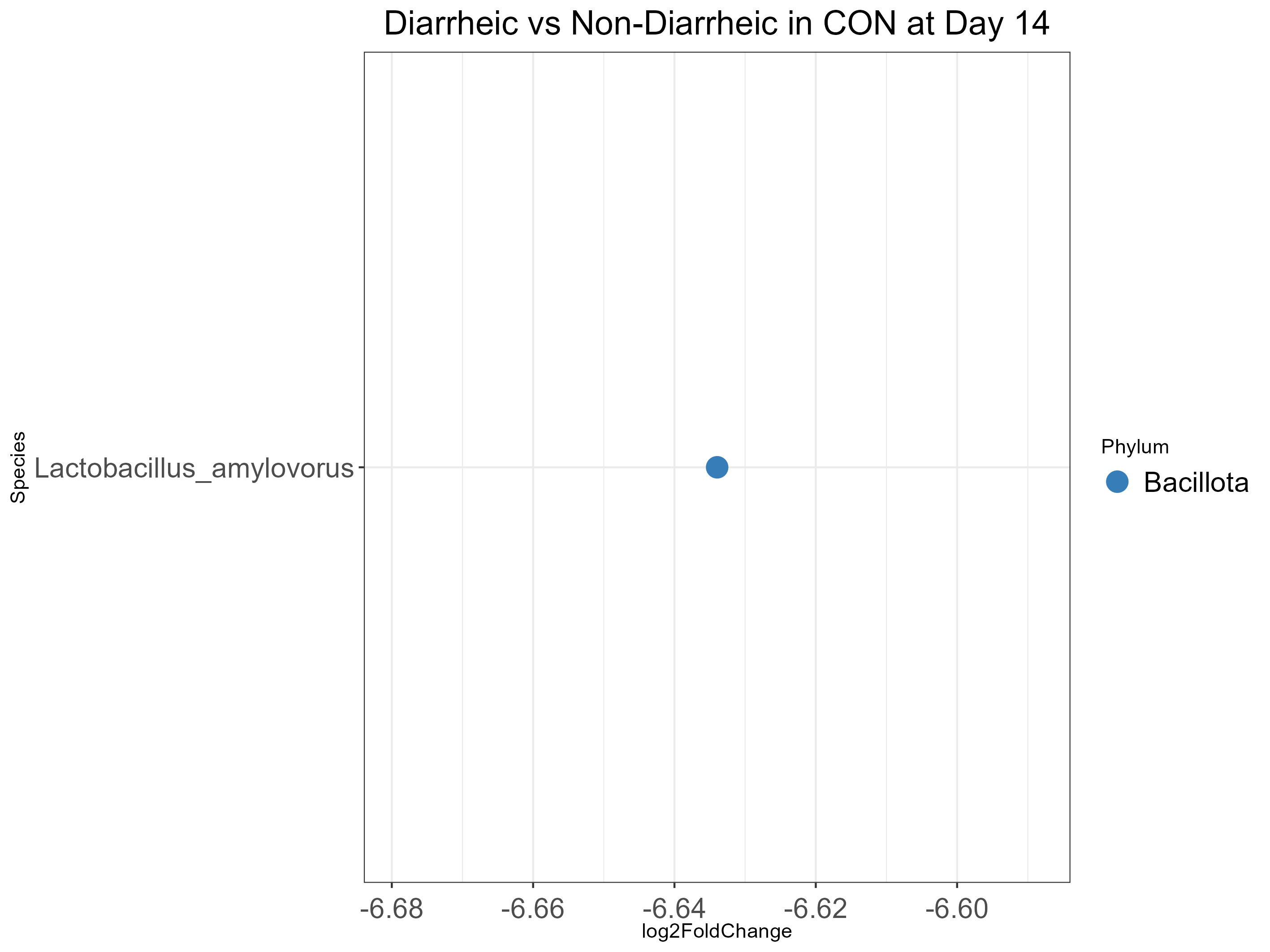

Supplement: Supplementary file 1 [file microorganisms-13-01810-s001.zip › FigS5C_Diarrheic_CON_D14.tiff]

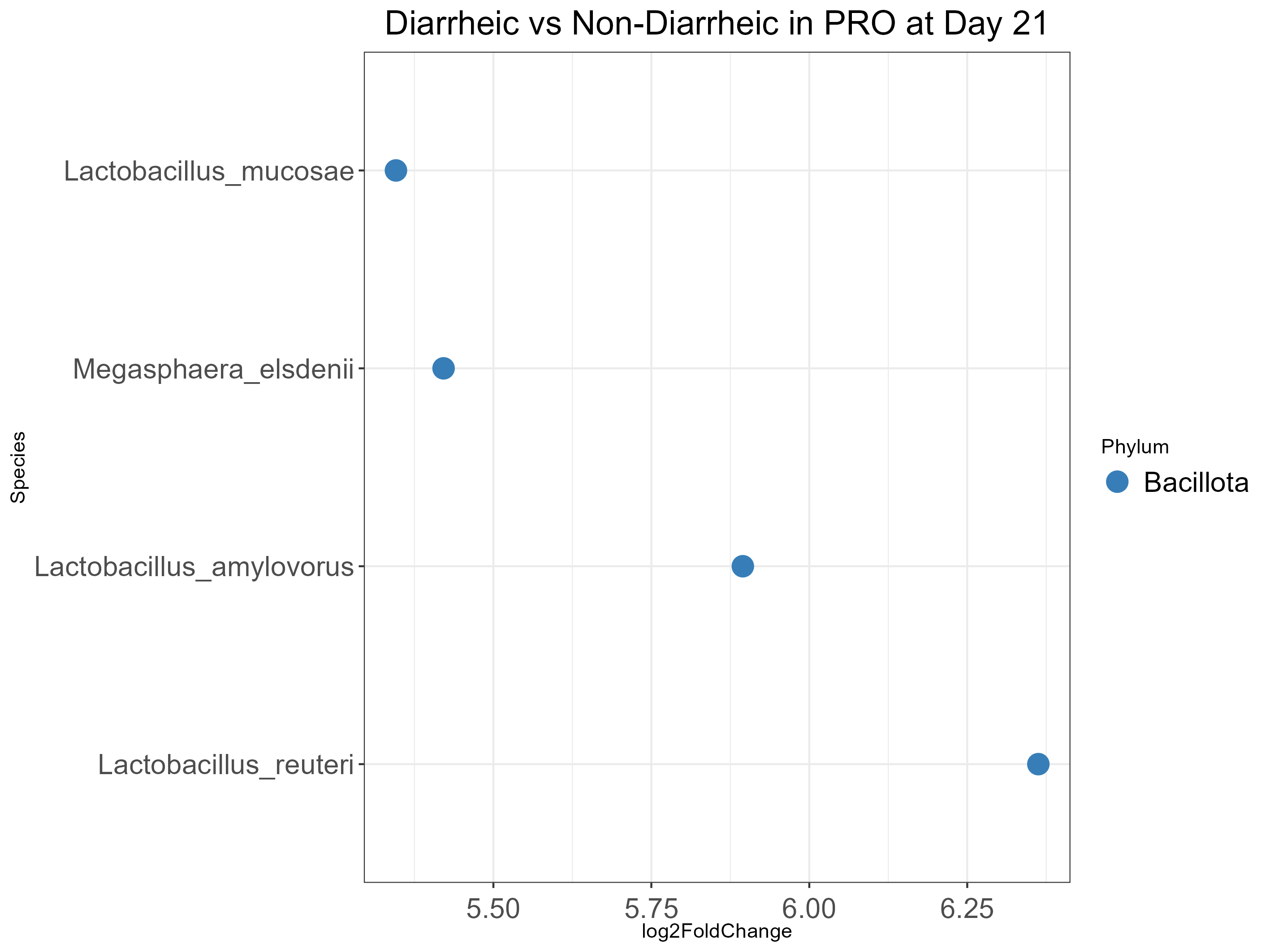

Supplement: Supplementary file 1 [file microorganisms-13-01810-s001.zip › FigS5D_Diarrheic_PRO_D21.tiff]

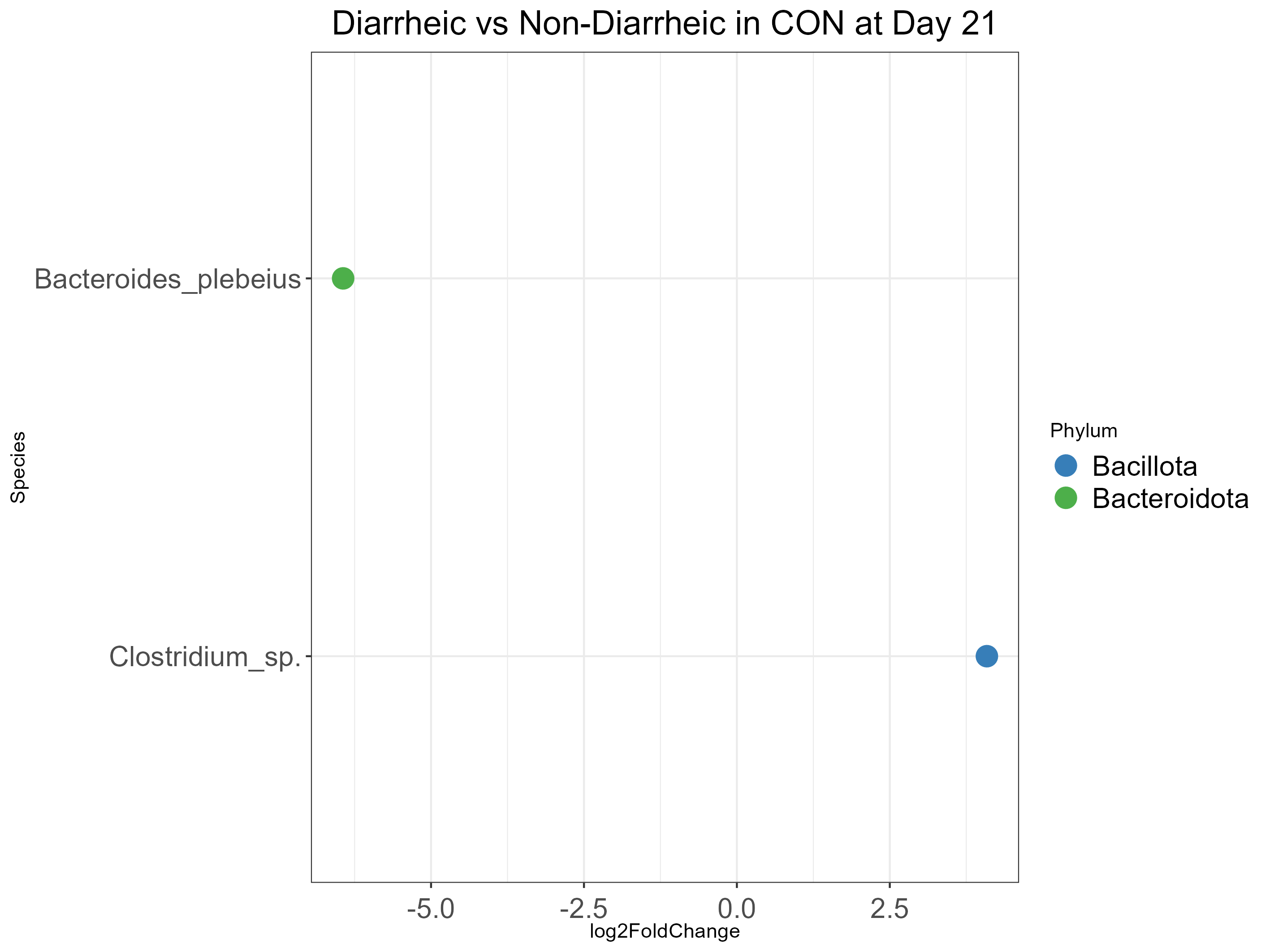

Supplement: Supplementary file 1 [file microorganisms-13-01810-s001.zip › FigS5E_Diarrheic_CON_D21.tiff]

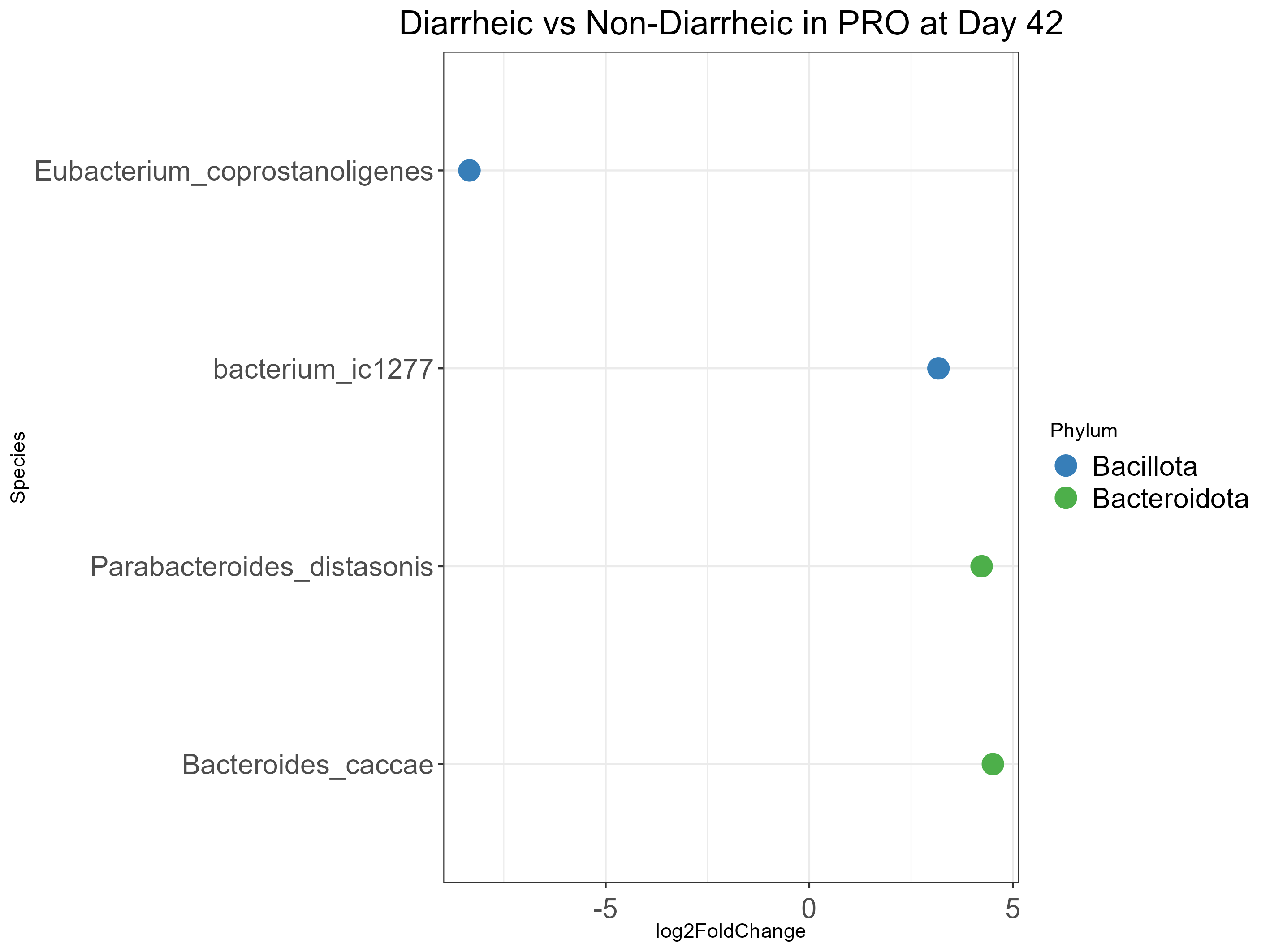

Supplement: Supplementary file 1 [file microorganisms-13-01810-s001.zip › FigS5F_Diarrheic_PRO_D42.tiff]

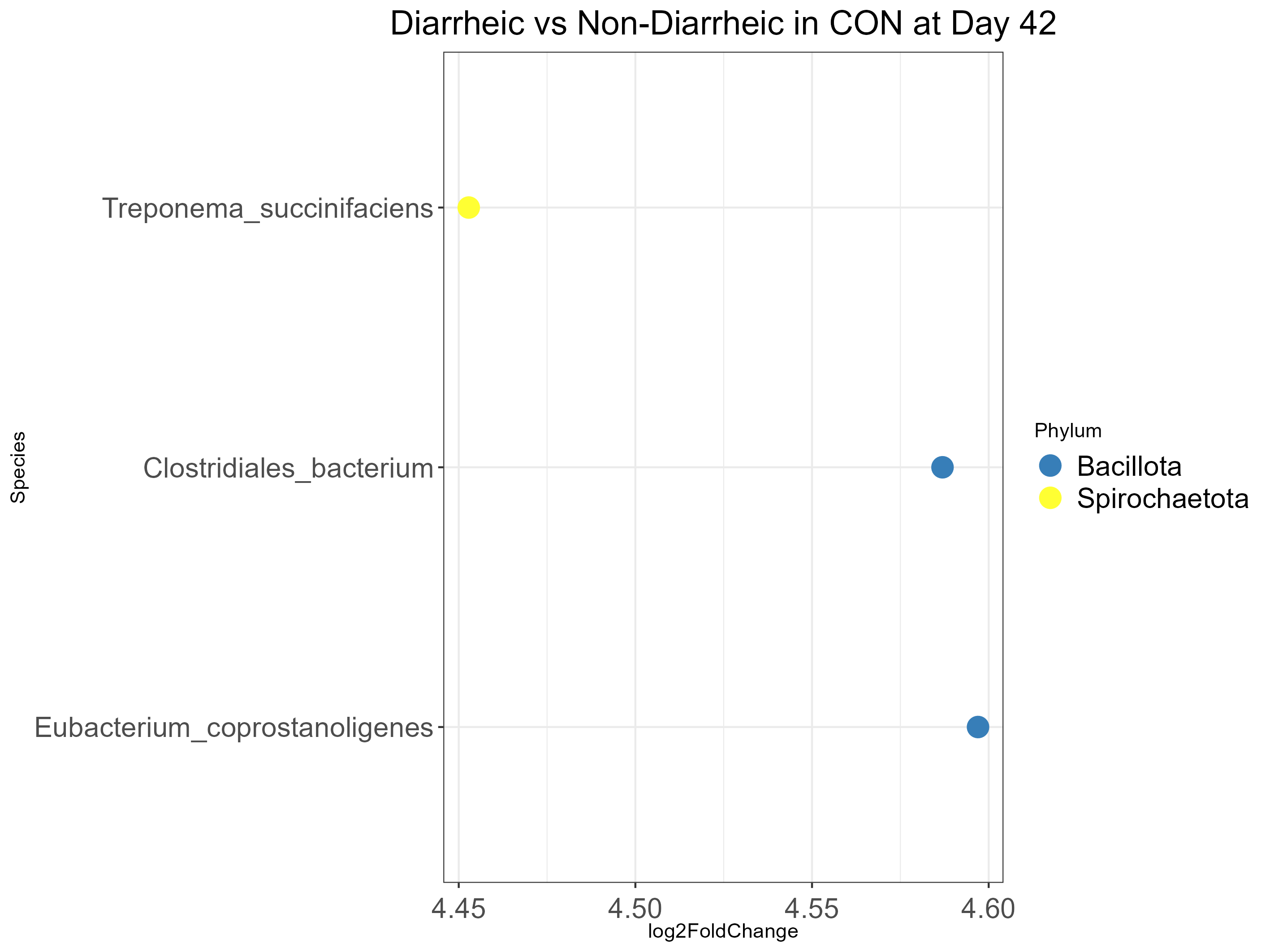

Supplement: Supplementary file 1 [file microorganisms-13-01810-s001.zip › FigS5G_Diarrheic_CON_D42.tiff]

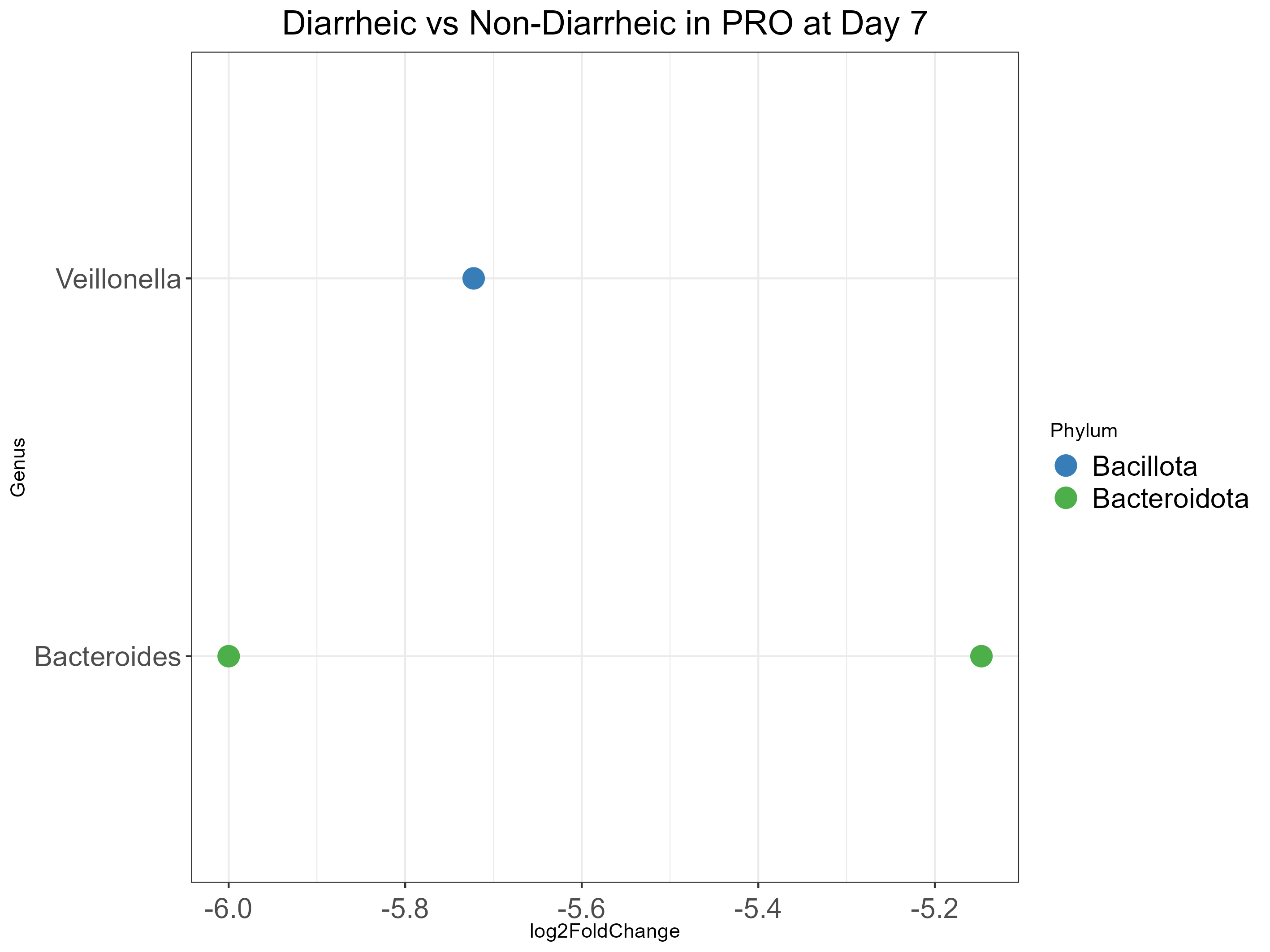

Supplement: Supplementary file 1 [file microorganisms-13-01810-s001.zip › FigS6A_Diarrheic_PRO_D7_Genus.tiff]

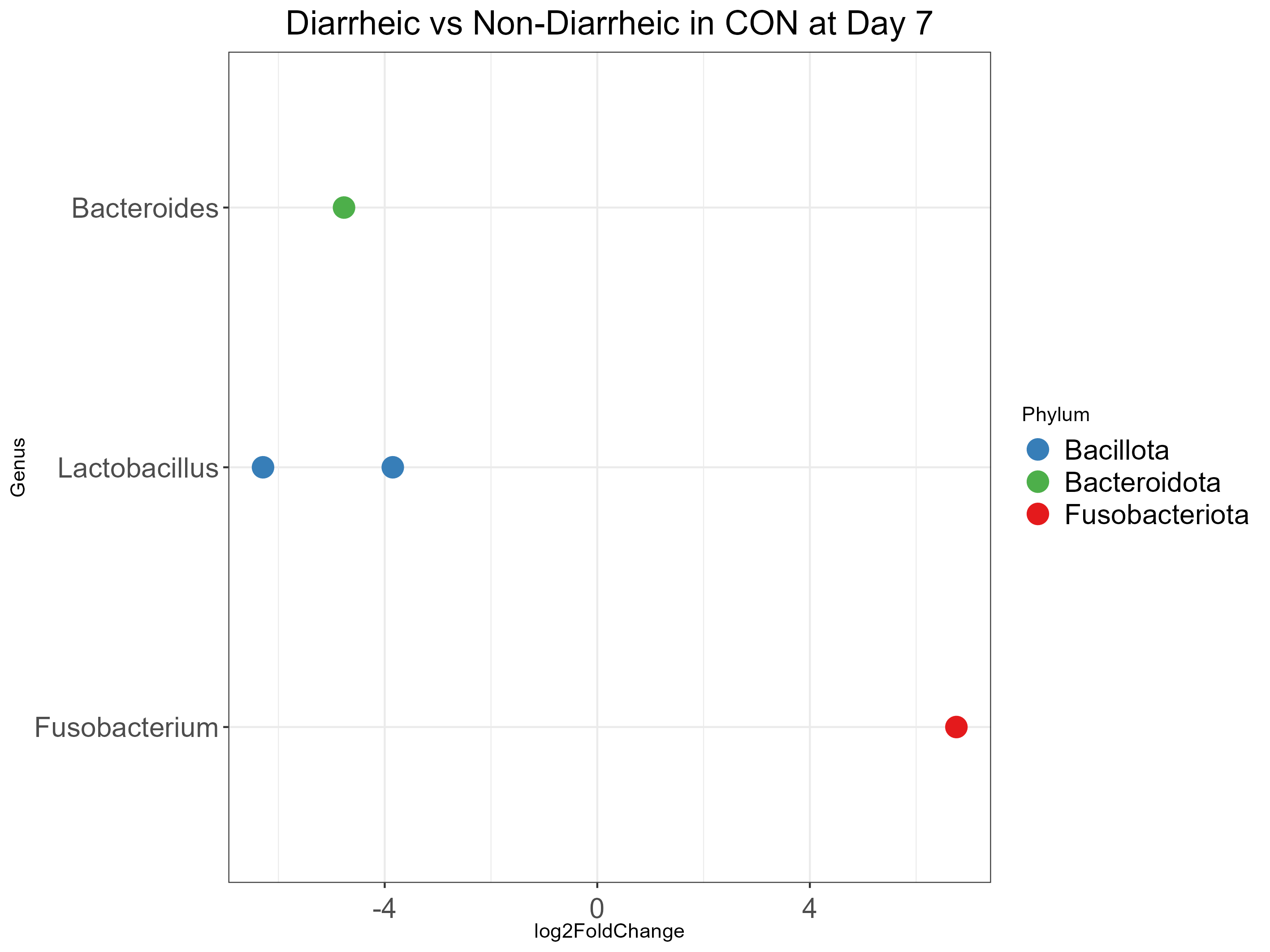

Supplement: Supplementary file 1 [file microorganisms-13-01810-s001.zip › FigS6B_Diarrheic_CON_D7_Genus.tiff]

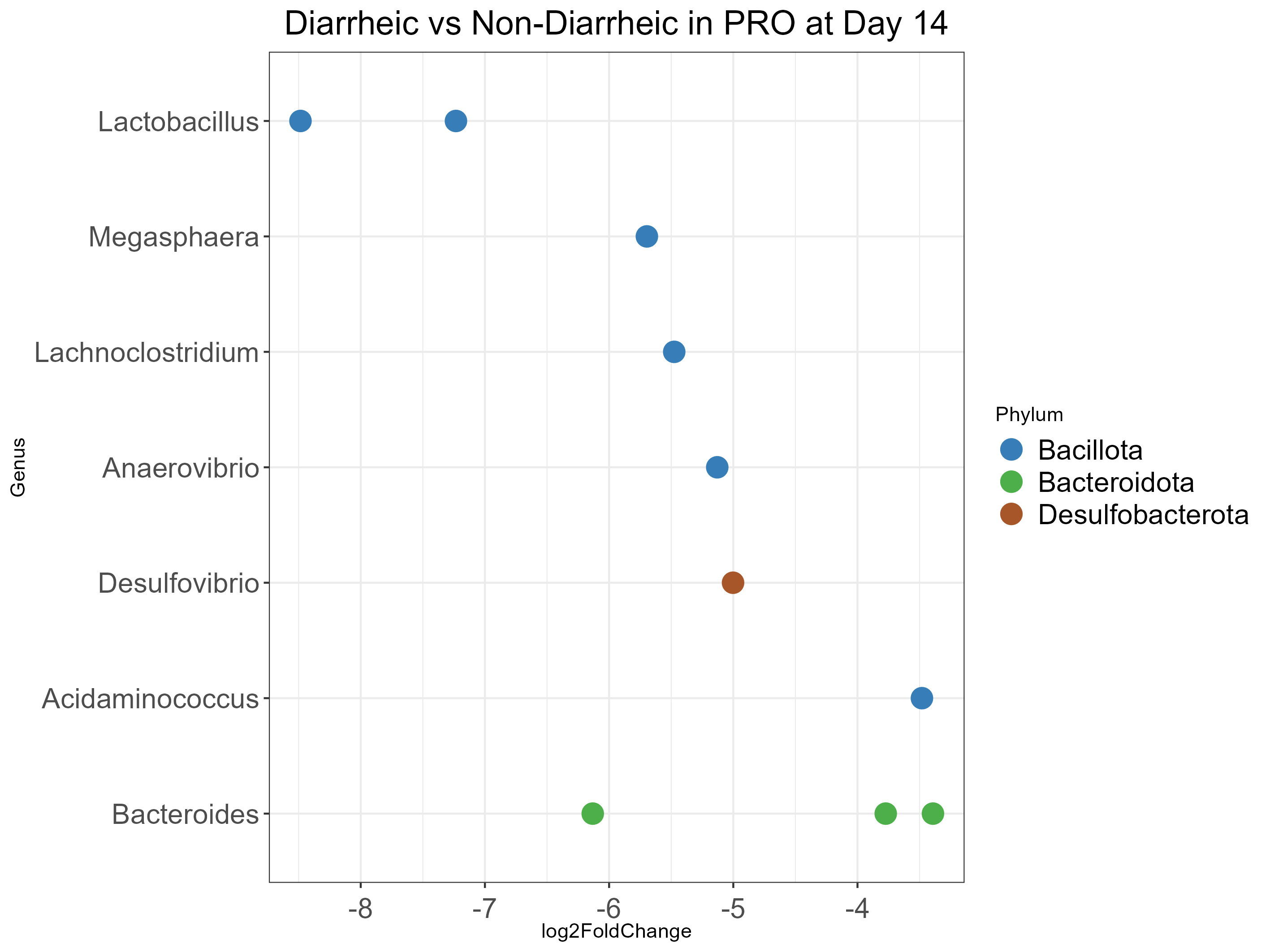

Supplement: Supplementary file 1 [file microorganisms-13-01810-s001.zip › FigS6C_Diarrheic_PRO_D14_Genus.tiff]

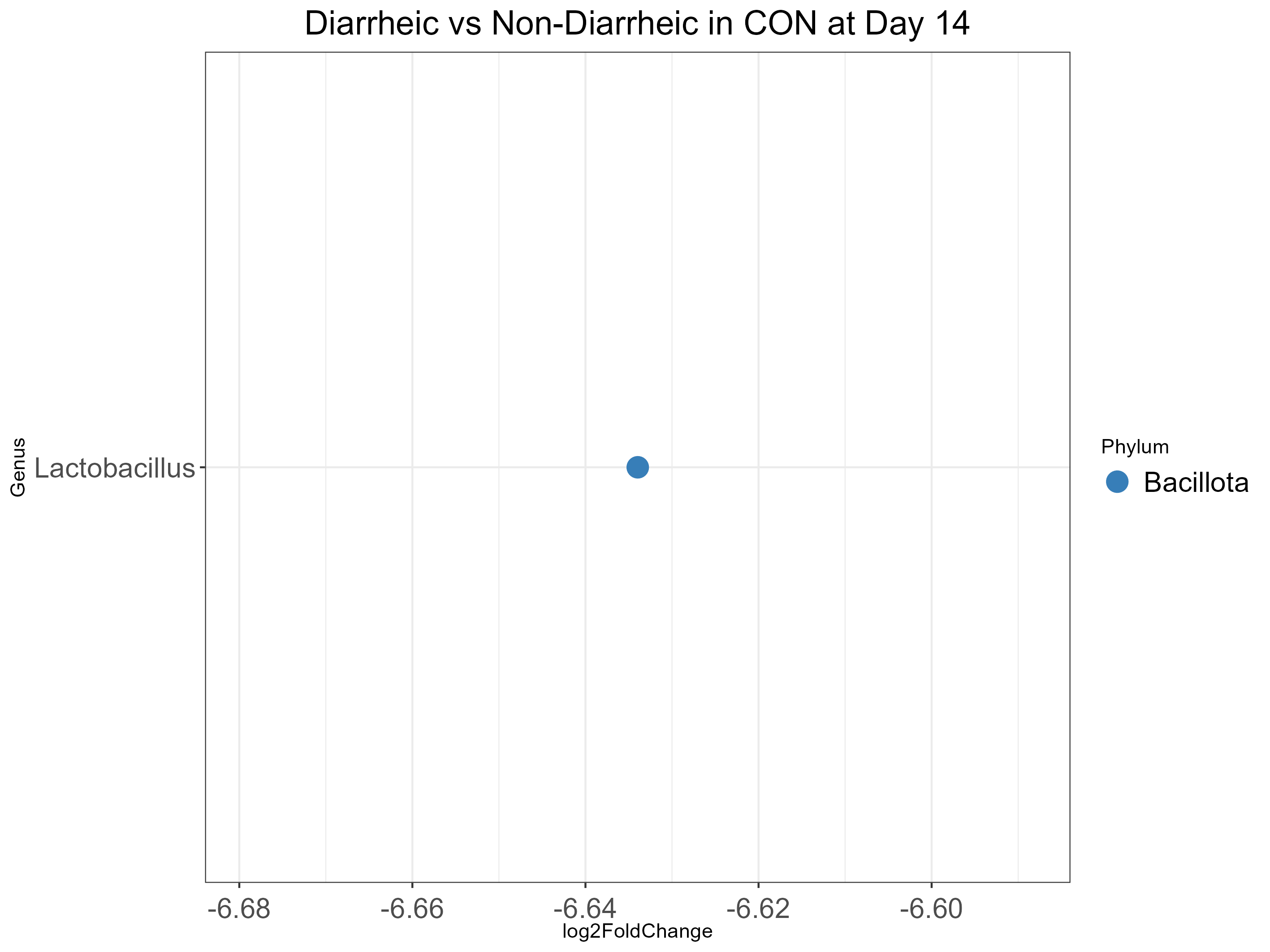

Supplement: Supplementary file 1 [file microorganisms-13-01810-s001.zip › FigS6D_Diarrheic_CON_D14_Genus.tiff]

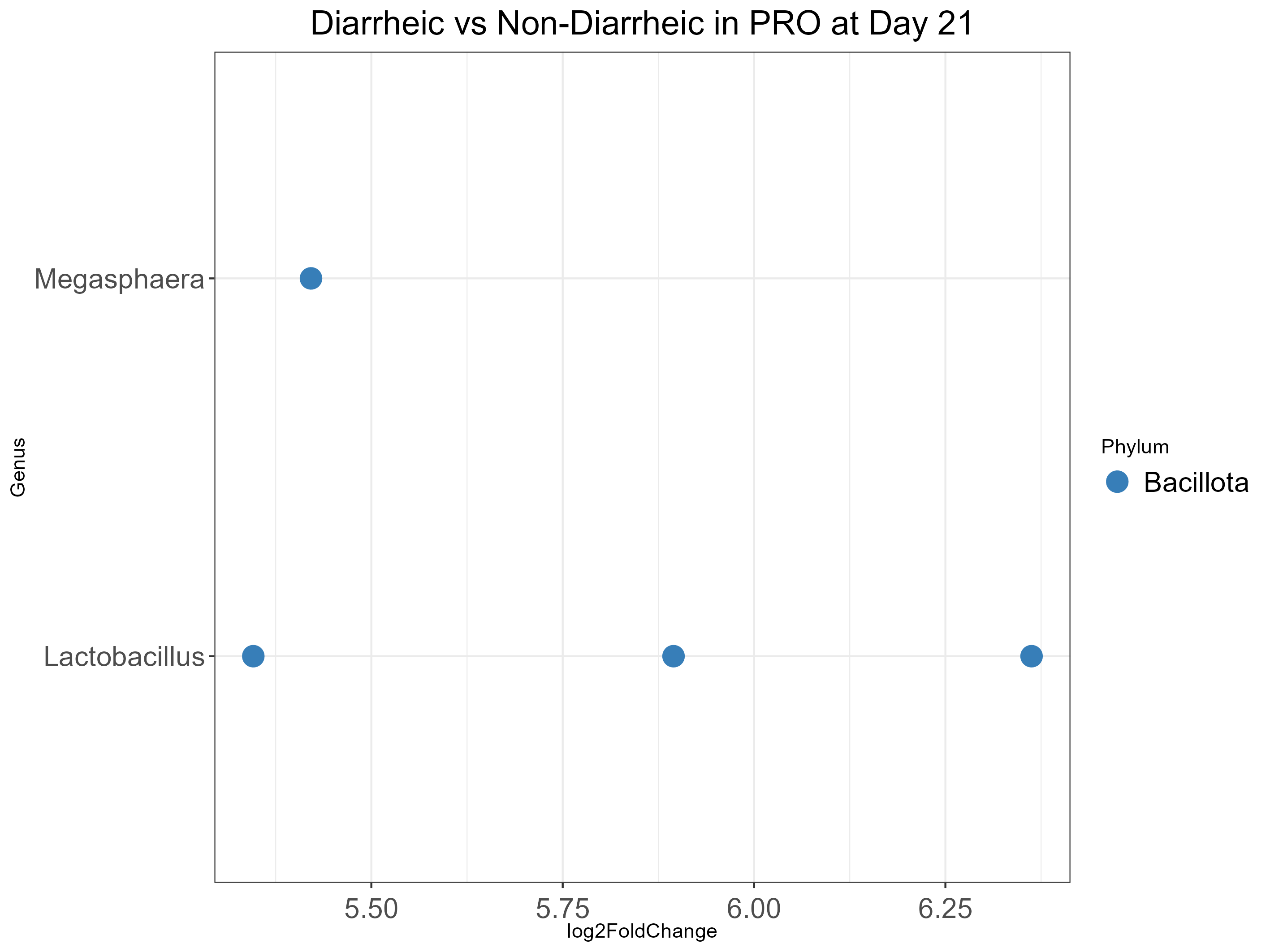

Supplement: Supplementary file 1 [file microorganisms-13-01810-s001.zip › FigS6E_Diarrheic_PRO_D21_Genus.tiff]

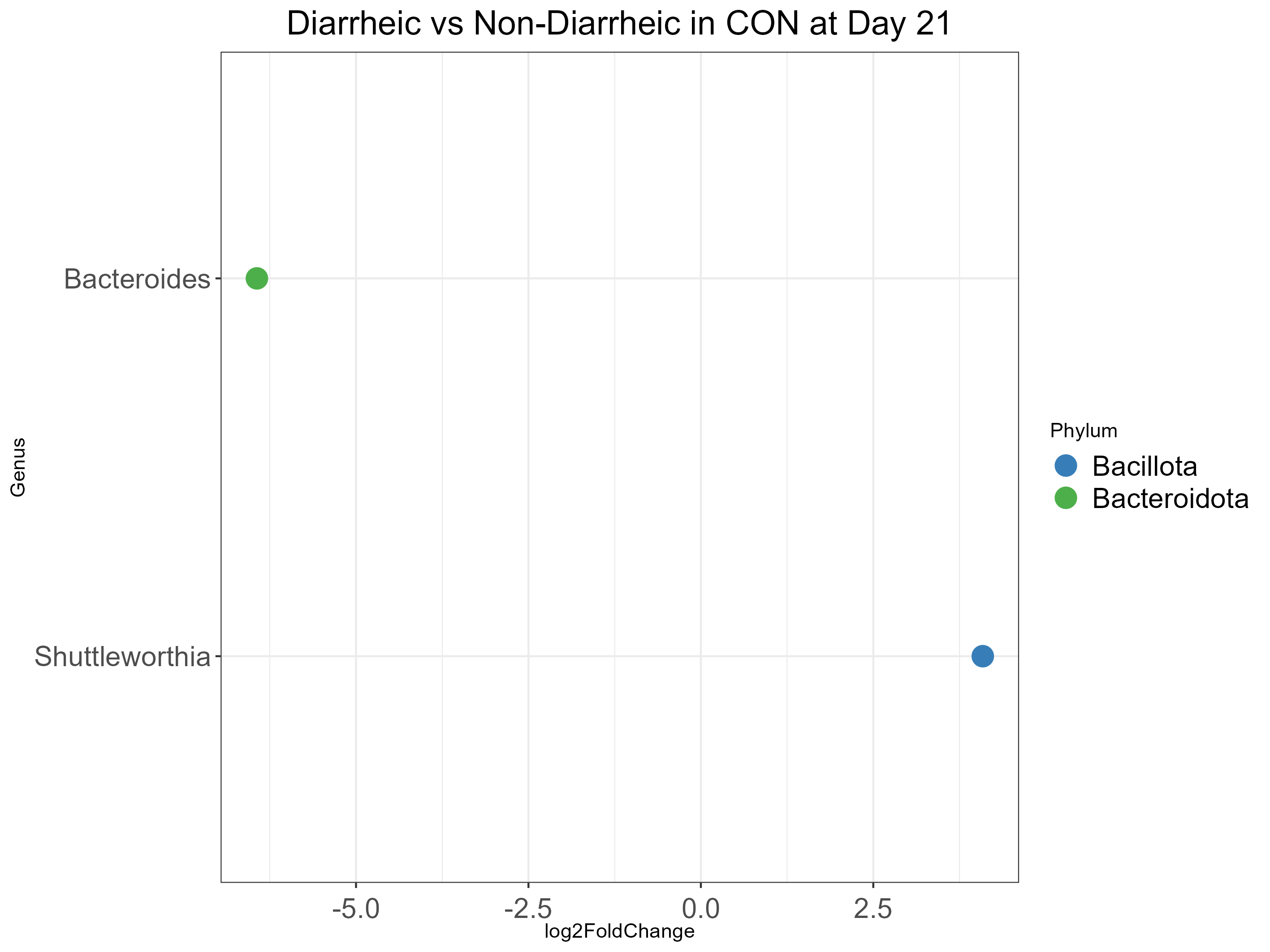

Supplement: Supplementary file 1 [file microorganisms-13-01810-s001.zip › FigS6F_Diarrheic_CON_D21_Genus.tiff]

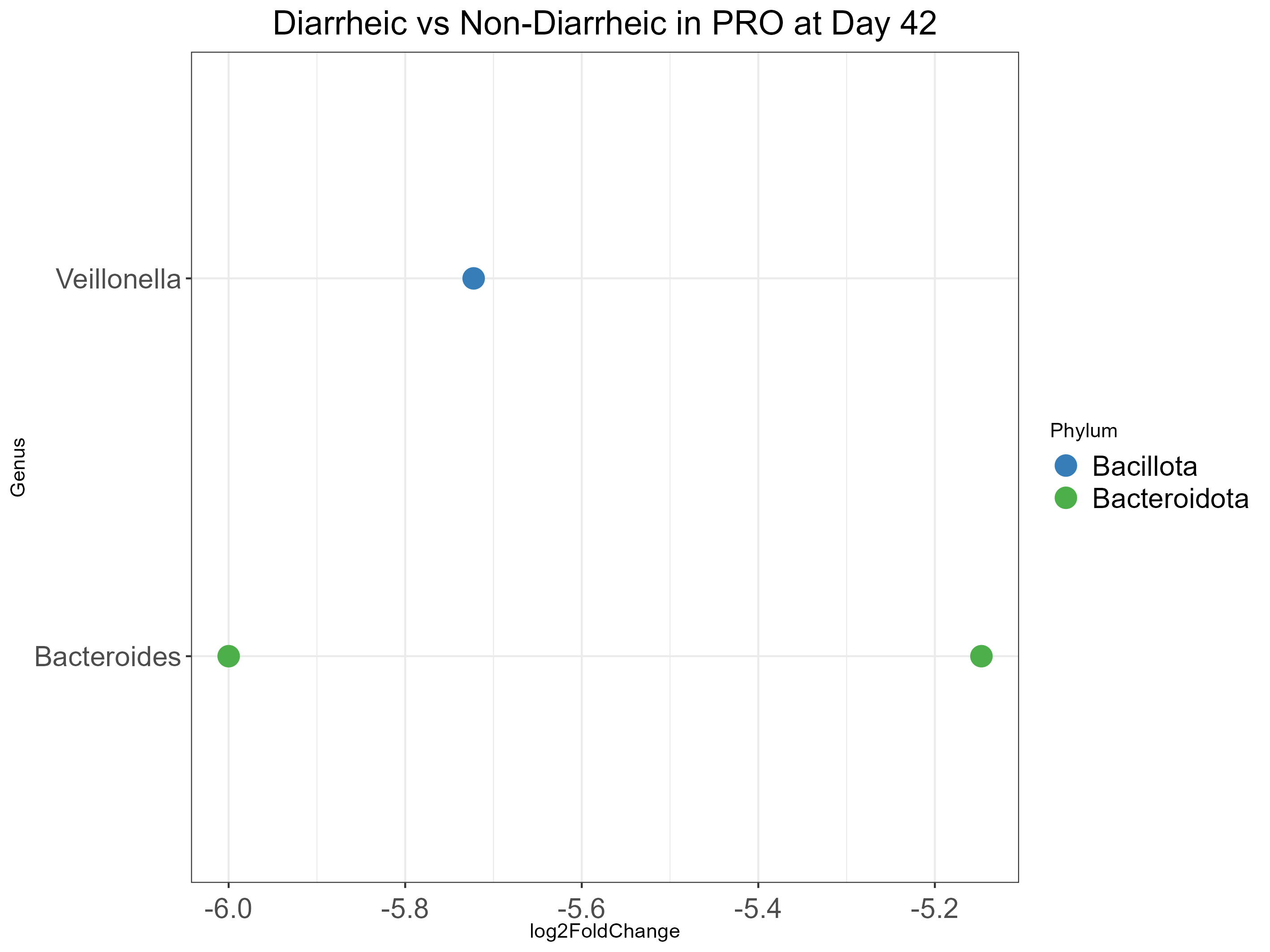

Supplement: Supplementary file 1 [file microorganisms-13-01810-s001.zip › FigS6G_Diarrheic_PRO_D42_Genus.tiff]

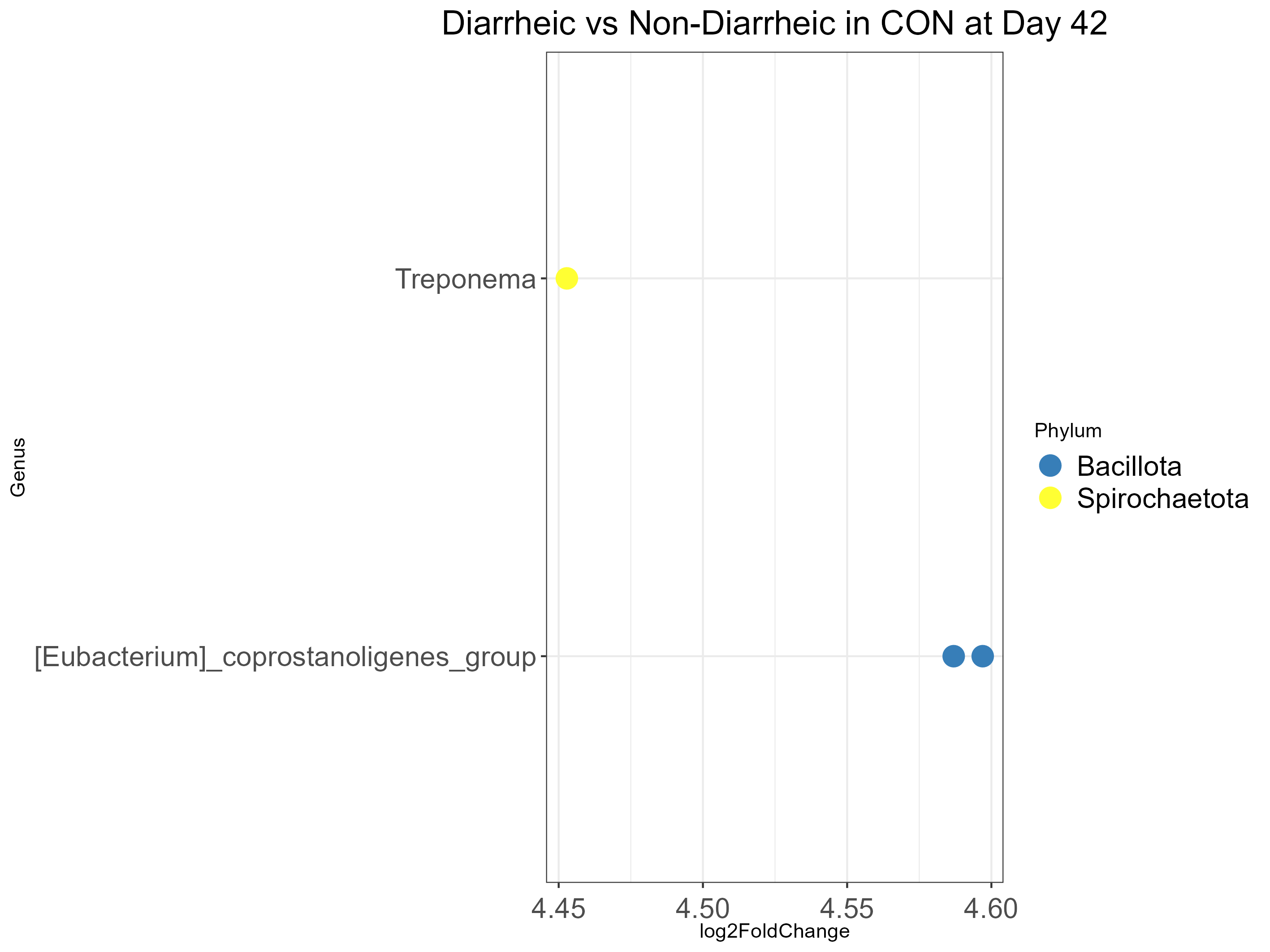

Supplement: Supplementary file 1 [file microorganisms-13-01810-s001.zip › FigS6H_Diarrheic_CON_D42_Genus.tiff]
